# Reliability Evaluation of RSrSF-LoRa and LoRaWAN for Dense Industrial IoT Networks in a Smelter Environment

**DOI:** 10.3390/s26165216

**Published:** 2026-08-17

**Authors:** Sonile K. Musonda, Musa Ndiaye, Zilole Simate, Hastings M. Libati, Adnan M. Abu-Mahfouz

**Affiliations:** 1 Department of Electrical Engineering, Copperbelt University, Kitwe 10101, Copperbelt, Zambia; sonile@cbu.ac.zm; 2 Department of Electrical and Computer Engineering, University of Namibia, JEDS Campus, Ongwediva 15006, Oshana, Namibia; mndiaye@unam.na; 3 Department of Computer Science, Namibia University of Science and Technology, Windhoek 9000, Khomas, Namibia; zsimate@nust.na; 4 Department of Computer Science, Copperbelt University, Kitwe 10101, Copperbelt, Zambia; libati@cbu.ac.zm; 5 Council of Scientific and Industrial Research, Pretoria 0083, Gauteng, South Africa; 6 Department of Electrical, Electronic and Computer Engineering, University of Pretoria, Pretoria 0002, Gauteng, South Africa

**Keywords:** RSrSF-LoRa, LoRaWAN, Industrial Internet of Things (IIoT), spreading factor reservation, packet delivery ratio (PDR), energy consumption

## Abstract

Industrial Internet of Things (IIoT) systems are increasingly deployed in heavy industrial facilities to support real-time monitoring, automation, and safety-critical operations. Long-range low-power communication technologies such as LoRaWAN are widely used for large-scale sensor connectivity due to their long communication range and energy efficiency. However, dense industrial deployments may experience reduced communication reliability due to network congestion, packet collisions, and challenging propagation conditions caused by metallic infrastructure and electromagnetic interference. To address these limitations, this paper proposes RSrSF-LoRa, an integrated implementation of the reserved spreading factor (rSF) mechanism within the RS-LoRa framework (lightweight scheduling), designed to improve reliability for critical traffic. The study presents a comparative performance evaluation of RSrSF-LoRa and LoRaWAN in a smelter IIoT deployment, assessing packet delivery ratio (PDR), throughput, fairness, energy consumption and scalability under varying node densities and gateway configurations. Single-gateway scenarios with 100, 500, and 1000 nodes, and seven-gateway scenarios with 1000 and 2100 nodes, are evaluated as communication distances increase. The results indicate that while throughput and fairness remain comparable across approaches, RSrSF-LoRa improves alarm message reliability in dense single-gateway deployments, extending the acceptable PDR from 700 m to 900 m. Alarm nodes in RSrSF-LoRa consume slightly more energy due to reserved transmission, but overall energy consumption remains comparable. These findings provide design insights for reliable and energy-aware industrial IoT networks in smelter environments.

## 1. Introduction

Industrial Internet of Things (IIoT) systems are increasingly deployed in heavy industrial facilities to enable real-time monitoring, predictive maintenance, and safety-critical operations. Cuozzo et al. [1] suggest that IIoT systems require stringent reliability, latency, availability and timeliness. They state that reliability is measured as the percentage of successfully delivered packets within the required time frame, focusing on individual packet losses, while ensuring that packet losses are kept to a minimum to avoid application failure. Additionally, the study reports that the simultaneous connections of thousands of IoT devices can take advantage of small-cell or cell-free communication systems, in which cutting-edge low-power radio access techniques allow for higher coverage and capacity. Small-cell systems enable localised connectivity, providing wireless communication in specific zones of a factory or plant, which can help industrial networks meet scalability and flexibility requirements even in a dynamic environment with varying device densities. From our earlier work [2], where we conducted a survey on the mining industry in Zambia, we learnt that a copper smelter can have a smart mining infrastructure with 6000 sensors to cater for all processes at the smelter such as copper production, oxygen production, cobalt recovery, sulphuric acid production, water treatment and air quality monitoring. Different types of sensors are employed in various processes to monitor temperature, level, pressure, flow (for fuel, gases, and water) and equipment status. Ensuring reliable and energy-efficient wireless communication in these environments is challenging due to high node density, metallic infrastructure, electromagnetic interference, high temperatures, moving vehicles and long propagation distances. The authors of [3,4,5] report that metallic structures often lead to high path loss, signal attenuation and shadow fading. Failure to deliver critical alarm messages on time can lead to production downtime, safety and environmental hazards, or equipment damage, making reliable wireless communication a key requirement for industrial IoT networks.

Among low-power wide-area network (LPWAN) technologies, LoRaWAN has emerged as a popular solution due to its long-range coverage, low energy consumption, and ability to support large-scale deployments. However, in dense single-gateway deployments, LoRaWAN performance can be affected by packet collisions and channel congestion, which reduce the reliability of alarm and telemetry traffic. To address these limitations, alternative mechanisms such as lightweight scheduling (RS-LoRa) [6] have been proposed, as well as spreading factor reservation for prioritising critical traffic [7]. However, the effectiveness of such mechanisms in realistic industrial smelter environments, particularly under varying node densities and gateway architectures, remains largely unexplored.

To bridge this gap, this paper presents RSrSF-LoRa, a hybrid implementation of RS-LoRa with a reserved spreading factor mechanism, and evaluates its performance against conventional LoRaWAN in smelter IoT deployments. The study considers multiple network scenarios: single-gateway deployments with 100, 500 and 1000 nodes, and seven-gateway deployments with 1000 and 2100 nodes. Performance is assessed using key metrics including packet delivery ratio (PDR), throughput, fairness, scalability and energy consumption, with particular focus on scenarios where PDR variability and alarm node energy usage are significant. The simulation results demonstrate that RSrSF-LoRa improves the reliability of alarm communication in dense single-gateway deployments, extending reliable transmission distances from 700 m to 900 m, while achieving comparable performance in multi-gateway or lower-density scenarios.

The main contributions of this work are as follows:

1. We propose RSrSF-LoRa, a novel reliability-aware communication framework that extends RS-LoRa with a reserved spreading factor (rSF) mechanism to prioritize mission-critical alarm traffic while supporting concurrent telemetry communication in dense industrial IoT networks. To enable realistic evaluation, a comprehensive smelter-specific wireless mining channel model and differentiated alarm and telemetry end-device traffic models are developed, with all simulations done using the ns-3 LoRaWAN module (ns-3.30.1; nsnam, https://www.nsnam.org, accessed on 13 August 2026).
2. We provide design insights for large-scale Industrial IoT networks by identifying practical deployment scenarios where RSrSF-LoRa enhances communication reliability for critical industrial traffic without significant energy penalties.

The remainder of the paper is organized as follows. Section 2 reviews related work on LoRaWAN, RS-LoRa, and SF reservation strategies. Section 3 describes the system and mining channel model as well as the RSrSF-LoRa mechanism, while Section 4 outlines the implementation. Section 5 presents the performance evaluation and discussion. Finally, Section 6 concludes this paper and highlights future research directions.

## 2. Related Work

The related works cover studies that have been done on LoRaWAN in mining environments to determine its reliability and the feasibility of its use in mining applications, as well as specific studies aimed at improving its performance. Additional studies involve the performance of LoRaWAN in industrial environments and LoRaWAN reliability in general. Nessa et al. [8] evaluated the performance of LoRa and LoRaWAN technologies in an underground mine in the presence of different kinds of traffic and proposed a redundant retransmission-aided adaptive latency reduction protocol for low-latency communication where the ACK-timeout is adjusted based on the air time of the previous uplink transmission and contention stage. Simulation results demonstrated a significant improvement in the performance of the proposed system compared to LoRaWAN in terms of data extraction ratio (DER) and average transmission delay for emergency traffic. However, DERs for localisation and sensing data were degraded in the proposed protocol compared to LoRaWAN. A new MAC protocol based on time division multiple access (TDMA) is developed by the authors of [9] to provide better connectivity to the IIoT system in harsh underground environments using LoRa wireless technology, together with an algorithm to support roaming that manages slot assignments to eliminate collisions while minimising the number of needed slots for a given number of gateways. The proposed MAC protocol and experimental tests provide practical results, showcasing its viability as an attractive alternative solution to meet the challenges presented in the mining and tunneling industry.

Other studies in mining involve LoRa propagation and coverage measurements as well as development of propagation models in the underground mine environment. Branch et al. [10] took measurements of LoRa radio propagation in a selected drift segment of an underground gold mine for line-of-sight (LOS) and non-line-of-sight (NLOS) communication. They further developed foundation models for LoRa signal behavior in mining scenarios, focusing on the block cave mining method. The findings were that for LOS communication, path loss was well modeled by a power law with a path loss index of 1.25 and variability in signal strength using a log-normal distribution with a mean of 4.5 dB. NLOS over short distances was successfully modeled using Fresnel diffraction with a path loss of 2 and a free-space model. They also studied the effects of SF on signal strength, which indicated that SF has a variable impact on the received signal strength indicator (RSSI) and a more consistent effect on signal-to-noise ratio (SNR). Their findings gave a good understanding of LoRa behaviour in this environment, which would lead to the development of LoRa applications underground. In [11], the authors conducted a study involving design and deployment of a LoRa-based IIoT system for monitoring the underground coal mine at the shaft, roadway, and face region. In addition, a new data rate algorithm for mesh network topology is proposed to optimise the data rate and maximise the coverage distance with minimal repeaters. Implementation of the proposed algorithm achieves a 1.46 kbps data rate at SF9, a bandwidth of 125 kHz and transmitter power of 11 dBm while extending the battery life of the deployed end nodes. Deployed end nodes achieve a maximum of 180 m under diffused LOS conditions in the roadway region of the operational mine.

Additional studies consider performance evaluation of LoRaWAN in industrial environments. Mining is an industrial environment; thus, these studies provide options that can be considered for deployment in these environments. A study carried out by the authors of [7] determined the performance of LoRaWAN for handling telemetry and alarms in industrial applications in which the potential of using spreading factor allocation strategies to prioritise alarm messages in the indoor and open-field environment was investigated. System-level simulations were used to compare the proposed solution with a benchmark scenario where no alarm was considered. The results show that it is possible to achieve high reliability with reasonably low delay without significantly affecting the performance of regular links. Magrin et al. [12] evaluate the performance of LoRaWAN in industrial scenarios for IIoT end nodes providing monitoring and sensing information by utilising the 2.4 GHz frequency for the EU region. They also analyse higher-layer impacts of different LoRa physical (PHY) layer models using industrial channel models, considering confirmed/unconfirmed traffic and multi-gateway deployments. The implementation changes were made for the publicly available LoRaWAN module for Network Simulator 3 (ns-3). The results show that when utilizing the frequency 2.4 GHz, the transmission time in the LoRaWAN network improved by 80%, decreasing from 75 ms to 14 ms, and resulted in the best performance, with a packet delivery rate above 90% due to the extended bandwidth in the case of the massive machine-type communication (mMTC) scenario with thousands of devices deployed.

A few studies involved improving the reliability and scalability of LoRaWAN in non-mining and non-industrial environments. However, they provide useful insights that could benefit these environments. Reynders et al. [6] propose a new MAC layer, RS-LoRa, to improve the reliability and scalability of LoRa wide-area networks (LoRaWANs) and implemented it in ns-3, evaluating its performance through extensive simulations. RS-LoRa involves a two-step lightweight scheduling that starts with the gateway and then the end nodes. Through the lightweight scheduling, nodes are guided to select different SFs to increase network reliability and scalability. The results show that in a single gateway of 1000 nodes, RS-LoRa can reduce the packet error ratio of legacy LoRaWAN by 20%. Herrería-Alonso et al. [13] developed Longest First-Slotted Carrier-Sense Multiple Access with Collision Avoidance (LFS-CSMA), a new MAC mechanism that enhances the scalability of LoRaWAN by combining the well-known slotted Aloha and CSMA schemes. Longer frames are transmitted earlier within a given time slot, and then devices with short frames to be transmitted can check the channel availability before sending them and avoid collisions if they detect an ongoing transmission. Results indicate that LFS-CSMA causes far fewer collisions than traditional MAC mechanisms, thus improving the scalability of LoRaWAN networks significantly.

In the context of LoRaWAN communications for mining and industrial applications, our work stands out by examining the performance of legacy LoRaWAN and the designed hybrid MAC mechanism RSrSF-LoRa in a smelter environment, a surface mining environment dominated by metallic structures (obstacles), interference, and moving objects. Many studies on IIoT networks involving LoRaWAN in mining are currently focusing on the underground environment. This study therefore provides design insights for reliable and energy-aware industrial IoT networks in smelter environments while adding a new dimension to the existing LoRaWAN research in mining.

Table 1 presents a summary comparison of the related works discussed in this section and our work.

## 3. System Design

In this section, the system and mining channel model are presented together with the RSrSF-LoRa algorithm that integrates the spreading factor reservation strategy and lightweight scheduling (RS-LoRa). Industrial LoRa deployments commonly exhibit heterogeneous traffic patterns, combining periodic telemetry with sporadic, time-critical alarm events. RS-LoRa improves scalability through probabilistic spreading factor allocation and coordinated access, but just like regular LoRaWAN, it does not explicitly differentiate between traffic classes with distinct reliability requirements. The purpose of the design, on the one hand, is to give priority to alarms by reserving an SF for them while at the same time using lightweight scheduling to distribute the remaining spreading factors across telemetry end nodes. Thus, lightweight scheduling helps to prevent overcrowding-assigned telemetry nodes to an inappropriate SF, a challenge noted by Filho et al. [7], which impaired telemetry communication in their study involving strategic allocation of spreading factors for the transmission of alarms and telemetry in industrial environments. On the other hand, integrating SF reservation and lightweight scheduling is aimed at addressing the packet collisions caused by the Aloha MAC protocol, channel congestion, and capture effect. Therefore, the design of RSrSF-LoRa is motivated by the observation that alarm traffic, although infrequent, must be protected from contention caused by telemetry transmissions while at the same time ensuring that telemetry transmission is not impaired.

### 3.1. System Model

The ns-3 LoRaWAN module does not classify the end nodes as alarms and telemetry; they are just created generally as end nodes (EDs); thus, we create two types of EDs, namely telemetry (regular) nodes and alarms (event-triggered) nodes. Telemetry EDs transmit packets to the network server via the gateways periodically at intervals of 600 s, while alarm EDs generate packets according to a Poisson process with a mean inter-arrival time of 600 s. This means that the inter-arrival time is exponentially distributed with mean 600 s, expressed as

(1) $$\Delta T∼\exp\left(\lambda =\frac{1}{600}\right).$$

and transmission time, expressed as

(2) $$T_{n}=\sum_{i=1}^{n}\Delta T_{i}.$$

The packet sizes for telemetry and the alarms are 28 and 14 bytes, respectively. We model a mining environment that is typical for the copper smelter outdoor environment and create a LoRa wide-area network that implements our algorithm at the MAC layer. We assumed a case of the air-quality monitoring system in which the EDs are not only placed within the smelter area but also in areas away from the smelter within the mine and away from the mine into the city. Copper smelting normally results in the production of toxic gases, such as sulphur dioxide and carbon dioxide, which can affect humans and other living organisms. Thus, sulphur dioxide, for instance, is safely channeled to the acid plant for production of sulphuric acid. The air monitoring system thus serves the critical purpose of ensuring that there is 0% of sulphur dioxide in the air. The gateway(s) can be connected to the network server using a wired connection such as Ethernet (optical Fibre) or wireless using a cellular connection (i.e., 4G or 5G). The system uses a combination of lightweight scheduling and spreading factor reservation. The spreading factor reservation is used to reserve a more robust spreading factor for alarms at any given time.

#### 3.1.1. Lightweight Scheduling

Lightweight scheduling starts with the gateways that are also responsible for coordinating the whole process and then with the EDs. Each channel is scheduled by specifying the allowed transmission power and SF in each subframe. The gateway specifies the allowed transmission power of the nodes using its received signal strength (RSS) of uplink traffic. This information is relayed to the EDs using beacons from the gateway and gives the allowed RSS of each channel per subframe. The ED joining process is the same as that of legacy LoRaWAN. Each ED uses the information in the beacons to choose its own transmission parameters, namely, channel, SF, and transmission power. This process is performed at each end node in a distributed manner. Based on the study by Reyders et al. [6], different spreading factors are selected with different probabilities. This can be represented mathematically as follows:

Let S be the set of all LoRa spreading factors {7, 8, 9, 10, 11, 12};
$S_{i}\subseteq S$ be the set of allowed SFs on channel i (this is what is advertised in the beacon);
$W_{s}\geq 0$ be the selection weight (or preference) for SFs.

These weights encode the lightweight scheduling policy (fairness, load balancing, learning, etc.). The probability that a node selects spreading factor s on channel i is generally given as

(3) $$P_{r}\left\{SF=s|i\right\}=\frac{W_{s}}{\sum_{k\in S_{i}}W_{k}},s\in S_{i}.$$

$s\notin S_{i}$ is forbidden, so its probability is zero. The denominator in the expression ensures that the probabilities sum to 1 over $S_{i}$. This means that given the set of admissible spreading factors $S_{i}$ announced in the beacon for channel i, an end device selects SF $s\in S_{i}$ with a probability proportional to a weight $W_{s}$.

More specifically, to achieve the lowest packet error ratio, Rappaport et al. [14] states that the preference is the achieved data rate; therefore, the weights are replaced by the achieved data rates. The expression for the probability of selecting an SF s by an end node is therefore

(4) $$P_{r}\left\{SF=s|i\right\}=P_{s}=\frac{R_{s}}{\sum_{s\prime \in S_{i}}R_{s\prime }},\forall s\in S_{i}.$$

where $S_{i}$ is the set of allowed spreading factors on channel i (from the beacon),

$s\in S_{i}$ is a candidate spreading factor,
$R_{s}$ is achievable data rate when using spreading factor s,
$R_{s\prime }$ is achievable data rate for another SF $s^{\prime }\in S_{i}$, and we have $\sum_{s\in S_{i}}P_{s}=1$.

The achievable coding rate $R_{s}$ is in turn given by

(5) $$R_{s}=\frac{s.BW.CR}{2^{s}}.$$

where s∈{7,8,9,10,11,12}, BW is bandwidth, and CR is the coding rate index.

This means that lightweight scheduling enables EDs to select spreading factors probabilistically, assigning higher selection probability to SFs with higher achievable data rates, thereby reducing airtime and improving overall spectral efficiency.

**Reliability of Beacons:** Beacons are used for carrying downlink information and synchronising nodes. Once a node wakes up, it listens to the latest beacon and then adapts its clock to the gateway. To increase the reliability of beacons against collisions with beacons from other gateways and interference from other networks operating at the same frequency, they are transmitted on different channels. Additionally, different SFs are used to transmit them, so as to increase reception probability and to ensure that the first beacon is received by all end nodes, including those at the cell edge; it is sent using SF12. For a detailed illustration of this aspect together with channel assignment, refer to the study by the authors of [6].

The lightweight scheduling concept is being used in our work to help distribute spreading factors for telemetry nodes, a measure taken to prevent them from crowding in one inappropriate SF.

#### 3.1.2. Spreading Factor Reservation

Using the lightweight scheduling information, each alarm end node determines the SF to use for transmission, and based on all SF values estimated as adequate for the alarm nodes, the highest one is chosen and adopted as the reserved SF (rSF). Hence, all the alarm nodes transmitting at a particular instance will be allocated the rSF. The telemetry nodes will also use the lightweight scheduling information to determine the SF to use for transmission but will not be allocated the rSF unless it is the highest one, that is, SF12. To prevent telemetry nodes from choosing the rSF when it is less than SF12, they will choose another SF from the set of SFs available for the selected channel. For instance: If the set of allowed SFs for a channel = SF8, SF9, and SF10 and rSF is SF9, a telemetry node can choose either SF8 or SF10. Another example is if the set of allowed SFs for a channel = SF10, SF11, and SF12 and if rSF is SF11, a telemetry node can choose either SF10 or SF12. However, if from the lightweight scheduling a telemetry node is assigned to use SF12 and the rSF is SF12, it will maintain SF12. This concept is being used to enable reservation of SF for alarms that is more robust as a way of giving priority to alarm transmission.

#### 3.1.3. RSrSF-LoRa

Upon receiving the beacon message from the gateway specifying the allowed transmission parameters per channel for each subframe, the EDs will use this information to determine their applicable or own transmission parameters. The steps for both alarm and telemetry nodes are as follows:

**Alarm end nodes:**

1. Find the available channel by listening to the latest beacon.
2. Determine the channel by searching over all available channels.
3. Select one with the highest target RSS ($RSS_{HT}$) which is lower than the estimated RSS of the beacon from the gateway ($RSS_{B}$).
4. If the channel is found, a random SF is selected from $S_{i}$, where $S_{i}$ is the set of allowed SFs per channel,
  - Compare SFs selected by alarms for this subframe and assign the highest one to all alarms. This is the reserved SF (rSF).
  - Select the channel that has this rSF and matching estimated $RSS_{HT}$ of each alarm.
  Otherwise, if the channel is not found, the lowest SF (SF = 7) is selected, which means the alarm node is close to the gateway.
5. Compute the transmit power using this rSF and estimated $RSS_{HT}$.
6. Select a random time for transmission between beacons (before the arrival of the next beacon).

**Telemetry end nodes:**

1. Find the available channel by listening to the latest beacon.
2. Determine the channel by searching over all available channels.
3. Select one with the highest target RSS that is lower than the estimated RSS of the beacon from the gateway.
4. If the channel is found, a random SF is selected from $S_{i}$, where $S_{i}$ is the set of allowed SFs per channel.
  **Case 1:** If the selected SF = = rSF and rSF<12, choose SF from the set of remaining SFs, $S_{r}=S_{i}-\mathrm{rSF}$ and proceed to step 5.
  **Case 2:** If the selected SF = = rSF and rSF = = 12, maintain this SF and proceed to step 5.
  **Case 3:** If the selected SF! = rSF, proceed to step 5.
  Otherwise, if the channel is not found, the lowest SF is selected, which means that the end node is close to the gateway.
5. Calculate required transmit power based on the determined SF and target RSS. It is worth noting that an increase in SF by 1 brings an increase of 2.5 dB in the sensitivity of the gateway, according to the datasheet of the LoRa transceiver (SX1272/73, Semtech Corporation, Camarillo, CA, USA) [15].
6. Select time for transmission between beacons (before the arrival of the next beacon).

The algorithm is presented below.

Apart from using the combination of spreading factor reservation and lightweight scheduling, other techniques have been incorporated to enhance the reliability of transmissions. Emergency traffic (alarms) is retransmitted whenever an acknowledgement (ACK) is not received within the two reception windows, with a maximum of eight retransmission attempts. Non-emergency traffic (telemetry) does not have retransmissions as a tradeoff measure to reduce the traffic load in the network and energy consumption. Another technique used is spatial diversity, which benefits both alarm and telemetry transmission because more than one gateway is used, and normally, for LoRaWAN transmission, EDs use the gateway with the strongest signal. In our simulations, we employed seven gateways because the higher the number of gateways, the better the spatial diversity according to the authors of [7].

### 3.2. Analysis of Computational and Implementation Overhead

RSrSF-LoRa is intended for deployment on battery-powered, memory-constrained EDs; thus, it is vital to analyse its computational and implementation overhead relative to legacy LoRaWAN and RS-LoRa. In this subsection, the complexity of Algorithm 1 is presented along with associated overhead. The parameters that determine the computational costs are as follows: the number of uplink channels C=|I|=3, the number of spreading factors |S|=6 (SF7-12), the total number of end devices N, the number of alarms $N_{a}$, and the number of telemetry nodes $N_{t}=N-N_{a}$.**Algorithm 1** To Determine Transmission Parameters For Alarm and Telemetry End Node**Input:** $N_{id}$, $P_{b}$, *I*, $T_{s}$, $S_{th}$, $S_{i}$, $P_{i}^{\prime }$, $S_{a}$**Output:** $C_{n}$, $S_{n}$, $S_{r}$, $P_{n}$, $T_{n}$  1:**if** $N_{id}=\mathrm{alarm}$ **then**  2:      $P_{tmp}\leftarrow 0$                                                                                  ▹$P_{tmp}$: a temporary variable  3:      Flag_Cn←false  4:      Flag_Sf←false  5:      **for** i∈I **do**  6:            **if** $P_{tmp}<P_{i}^{\prime }<P_{b}$ **then**  7:                  $C_{n}\leftarrow i$                                                                                            ▹ Select channel  8:                  $P_{tmp}\leftarrow P_{i}^{\prime }$  9:                  Flag_Cn←true10:            **end if**11:      **end for**12:      **if** Flag_Cn=true **then**13:            $S_{ai}\leftarrow \mathrm{randomly}\;\mathrm{choose}\;\mathrm{SF}\;\mathrm{from}\;S_{Cn}$14:            $S_{r}\leftarrow S_{ah}$                                                                      ▹ Highest SF from $S_{a}$ is reserved15:            Flag_Sf←true16:            Flag_Cn←false17:            **for** i∈I **do**18:                  **if** $S_{r}\;\&\;P_{tmp}$ **then**19:                       $C_{n}\leftarrow i$20:                       Flag_Cn←true21:                  **end if**22:            **end for**23:            **if** Flag_Cn=true **then**24:                  $P_{n}\leftarrow P_{tmp}+P_{pathloss}-2.5S_{n}+P_{offset}$25:            **else**26:                  $S_{r}\leftarrow 7$27:                  $P_{n}\leftarrow 0\;\mathrm{dBm}$28:                  $C_{n}\leftarrow {\mathrm{argmax}}_{i\in I}P_{i}^{\prime }$29:            **end if**30:            $T_{p}\leftarrow \mathrm{time}\text{-}\mathrm{of}\text{-}\mathrm{flight}\;\mathrm{of}\;\mathrm{packet}\;\mathrm{with}\;S_{r}$31:            $T_{n}\leftarrow \mathrm{rand}\left(0,T_{s}-T_{p}\right)$32:      **end if**33:**end if**34:**if** $N_{id}=\mathrm{telem}$ **then**35:      $P_{tmp}\leftarrow 0$36:      Flag_Cn←false37:**end if**38:**for** i∈I **do**39:      **if** $P_{tmp}<P_{i}^{\prime }<P_{b}$ **then**40:            $C_{n}\leftarrow i$41:            $P_{tmp}\leftarrow P_{i}^{\prime }$42:            Flag_Cn←true43:      **end if**44:**end for**45:**if** Flag_Cn=true **then**46:      $S_{n}\leftarrow \mathrm{randomly}\;\mathrm{choose}\;\mathrm{SF}\;\mathrm{from}\;S_{Cn}$47:      **if** $S_{n}=S_{r}\;\&\;S_{r}<12$ **then**48:            $S_{n}\leftarrow \mathrm{randomly}\;\mathrm{choose}\;\mathrm{SF}\;\mathrm{from}\;\left[S_{Cn}-S_{r}\right]$49:      **end if**50:      $P_{n}\leftarrow P_{tmp}+P_{pathloss}-2.5S_{n}+P_{offset}$51:**else**52:      $S_{n}\leftarrow 7$53:      $P_{n}\leftarrow 0\;\mathrm{dBm}$54:      $C_{n}\leftarrow {\mathrm{argmax}}_{i\in I}P_{i}^{\prime }$55:**end if**56:$T_{p}\leftarrow \mathrm{time}\text{-}\mathrm{of}\text{-}\mathrm{flight}\;\mathrm{with}\;S_{n}$57:$T_{n}\leftarrow \mathrm{rand}\left(0,T_{s}-T_{p}\right)$

#### 3.2.1. Time Complexity of Algorithm

1. **Channel search:** Scans the available channels once, performing two comparisons and at most three assignments per iteration. Therefore, the cost is Θ(|I|).
2. **Selection of spreading factor:** The weighted selection of Equation (4) is implemented and is achieved by inverse transform sampling, which requires determination of the achievable rate $R_{s}$ in Equation (5) for each admissible s, accumulation of the normalised sum, and a linear search of the resulting cumulative distribution. This gives the cost Θ(|S|) that dominates the per sub-frame cost.
3. **RSrSF-LoRa mechanism:**
  - Alarms: Locating the channel carrying the reserved spreading factor adds a second cost of Θ(|I|)
  - Telemetry: The collision test and resampling executed when the drawn SF coincides with the reserved SF and rSF<12 costs another Θ(|S|).

Table 2 gives the corresponding operation counts.

The sum of the contributions outlined above gives the per subframe ED complexity as

(6) $$T_{ED}=\Theta \left(\left|I\right|+\left|S\right|\right)$$

for both traffic classes. |I| and |S| are fixed by the parameters in Table 3; therefore, $I_{ED}$ is bounded by a constant in practice. Therefore, the complexity of the end device is in the same asymptotic class as RS-LoRa, with the reservation mechanism contributing an additive constant rather than a change in growth. It contributes 27 operations out of the 77 performed by an alarm ED and 45 of the 115 operations performed by a telemetry ED.

#### 3.2.2. Memory Complexity

The beacon-derived allowed SF set and the target RSS set are maintained by the end device, which requires Θ(|I||S|) storage, and this is inherited from RS-LoRa. RSrSF-LoRa thus adds the following: the traffic class (1 bit), reserved spreading factor (3 bits), a retransmission counter with a maximum of eight attempts (4 bits), an acknowledgment flag (1 bit), and one timer handle. This gives an incremented memory complexity Θ(1) equal to 8 bytes per device at most.

The per-subframe reserved-SF register at the gateway requires only 1 byte and an acknowledgment context of approximately $6N_{a}$ bytes, yielding the $\Theta (N_{a})$ gateway state. In the case of the dense single-gateway scenario where N=1000 and $N_{a}=20$, this is approximately 121 bytes. These values are negligible compared to the several kilobytes of RAM available on microcontrollers normally paired with $SX127_{x}$-class transceivers.

#### 3.2.3. Comparison with Alternative Mechanisms

The results obtained above for RSrSF-LoRa are compared with the reliability mechanisms reviewed in Section 2. LFS-CSMA [13] and TDMA MAC [9] have structurally higher costs required to achieve determinism. The former requires carrier sensing before every transmission, which demands an additional on-time receiver, an energy cost absent from purely scheduled schemes, while the latter requires per-slot assignment. RSrSF-LoRa retains the Θ(1) per-device cost of RS-LoRa while adding traffic differentiation. Table 3 summarises the complexity comparisons of the reliability schemes.

#### 3.2.4. Implementation Overhead

RSrSF-LoRa is implemented within the MAC sublayer, and, relative to RS-LoRa, this introduces only reserved SF selection, reserved SF comparison, optional channel selection, traffic classification, one additional transmit power calculation, and alarm retransmission. It does not change the physical layer, and the mining channel model is shared unchanged with the LoRaWAN baseline.

The practical consequence is that there is no change in modulation, transceiver configuration, or radio front-end behaviour. Implementation overheads are negligible, and RSrSF-LoRa, in principle, has the potential of being deployable on existing $SX127_{x}$-based hardware as a firmware update rather than a hardware replacement. However, the mechanism requires coordinated updates at both the gateways and the end devices, and is therefore an extension of RS-LoRa rather than an interoperable drop-in replacement for the unmodified LoRaWAN deployments.

### 3.3. Channel, Noise and Interference Model

The smelter environment is characterized by dense metallic structures, large obstructions, and significant electromagnetic interference. The authors of previous studies on industrial wireless channels report high path loss exponents, strong log-normal shadowing, severe multi-path fading, and elevated noise floors [3,4,16]. Given the similarity, and, in many aspects, increased harshness of smelters compared to underground mines and heavy industrial plants as indicated by Qaraqe et al. [17], the adopted channel model consisting of log-distance path loss, log-normal shadowing, Nakagami fading, and increased noise floor is well justified. In addition to static propagation impairments, industrial environments such as smelters contain mobile metallic objects including trucks, cranes, and forklifts. The authors of [18,19] report that these moving objects act as dynamic scatterers and blockers, causing time-varying shadowing and multi-path fading. To capture this non-stationary interference behavior, a way point mobility model was used to represent vehicle motion along predefined operational paths, which is a widely accepted abstraction for industrial mobility, as stated by Camp et al. [20]. Raza et al. [21] indicates that this approach allows realistic modeling of mobility-induced interference effects on long-duration LoRa transmissions.

All LoRa end devices considered in this work are stationary and deployed within and around a heavy-industrial smelter environment. The wireless channel is modeled as a composite propagation process that captures large-scale attenuation, shadowing, small-scale fading, receiver noise, and dynamic interference generated by moving mining vehicles.

#### 3.3.1. Propagation Effects

The large-scale path loss between a transmitting device i and a receiving device j, separated by a fixed distance $d_{ij}$, is modeled using a log-distance formulation:

(7) $$PL(d_{ij})=PL\left(d_{0}\right)+10n\log_{10}\left(\frac{d_{ij}}{d_{0}}\right),$$

where, $d_{0}$ = 1 m is the reference distance, $PL(d_{0})$ = 46.67 dB is the reference loss, and n≥3.5 is the path loss exponent. Studies by the authors of [22,23] report that this exponent reflects the severe attenuation caused by dense metallic structures such as furnaces, cranes, and ducts commonly found in smelters.

The authors of [14,16] show that shadowing due to large static obstructions is modeled as a log-normal random variable with standard deviation 6 dB, consistent with values reported for dense industrial environments, and is applied as an additive loss term to the large-scale path loss.

(8) $$X_{\sigma }∼N\left(0,\sigma^{2}\right),$$

where N denotes the normal (Gaussian) distribution.

The authors of [24,25,26] report that small-scale multipath fading is modeled using a Nakagami-m distribution with unit mean, which provides a flexible framework for representing a range of industrial multipath conditions without requiring explicit line-of-sight assumptions. The fading parameter m is distance-dependent, with Rayleigh fading (m = 1) at short distances and progressively milder fading at intermediate and larger distances (m = 1.5 and m = 2, respectively). This captures the transition from rich scattering near dense machinery to propagation dominated by a small number of strong reflected components over longer links. The resulting received signal power at device j is given by

(9) $$P_{r}=P_{t}-PL\left(d_{ij}\right)-X_{\sigma }+10\log_{10}\left(G_{f}\right),$$

where $P_{t}$ is the transmit power and $G_{f}$ denotes the Nakagami fading power gain.

#### 3.3.2. Receiver Noise

The authors of [27,28] indicate that the receiver noise is modeled as a constant noise floor applied at the physical layer. A noise power of *N* = −100 dBm is assumed, representing elevated thermal and electromagnetic interference conditions typical of heavy-industrial environments.

#### 3.3.3. Vehicle Induced-Interference

In addition to receiver noise, time-varying interference is introduced by heavy mining vehicles moving around the smelter. Studies by the authors of [14,29] report that these vehicles do not carry communication devices but act as dynamic sources of broadband electromagnetic interference due to their motion and electrical subsystems. Their motion is modeled using stochastic and way-point-based mobility patterns, resulting in a time-dependent aggregate interference power;

(10) $$I_{veh}\left(t\right)=\sum_{k=1}^{k}I_{k}\left(t\right),$$

where $I_{k}$(t) denotes the interference contribution of the k-th vehicle.

#### 3.3.4. Signal-to-Interference-and-Noise Ratio

Combining propagation effects, receiver noise, and vehicle-induced interference, the instantaneous signal-to-interference-and-noise ratio (SINR) at the receiver is expressed as

(11) $$SINR\left(t\right)=\frac{P_{r}\left(t\right)}{N+I_{veh}\left(t\right)}.$$

In decibel form,

(12) $$SINR_{dB}\left(t\right)=P_{t}-PL\left(d_{ij}\right)-X_{\sigma }+10\log_{10}\left(G_{f}\right)-10\log_{10}\left(N+I_{veh}\left(t\right)\right).$$

In (11), $P_{r}\left(t\right)$, N and $I_{veh}\left(t\right)$ are expressed in linear power quantities (mW). In (12), the same quantities are represented in logarithmic form, where powers are expressed in dBm and gains/losses in dB, yielding the SINR in dB.

Packet reception is determined by comparing the SINR to the spreading-factor-dependent LoRa demodulation threshold *γ*SF, as specified in LoRa physical-layer documentation and prior studies by the authors of [15,30]. A packet is successfully received if $SINR_{dB}\left(t\right)\geq \gamma SF$.

## 4. Implementation

**Figure 1:**
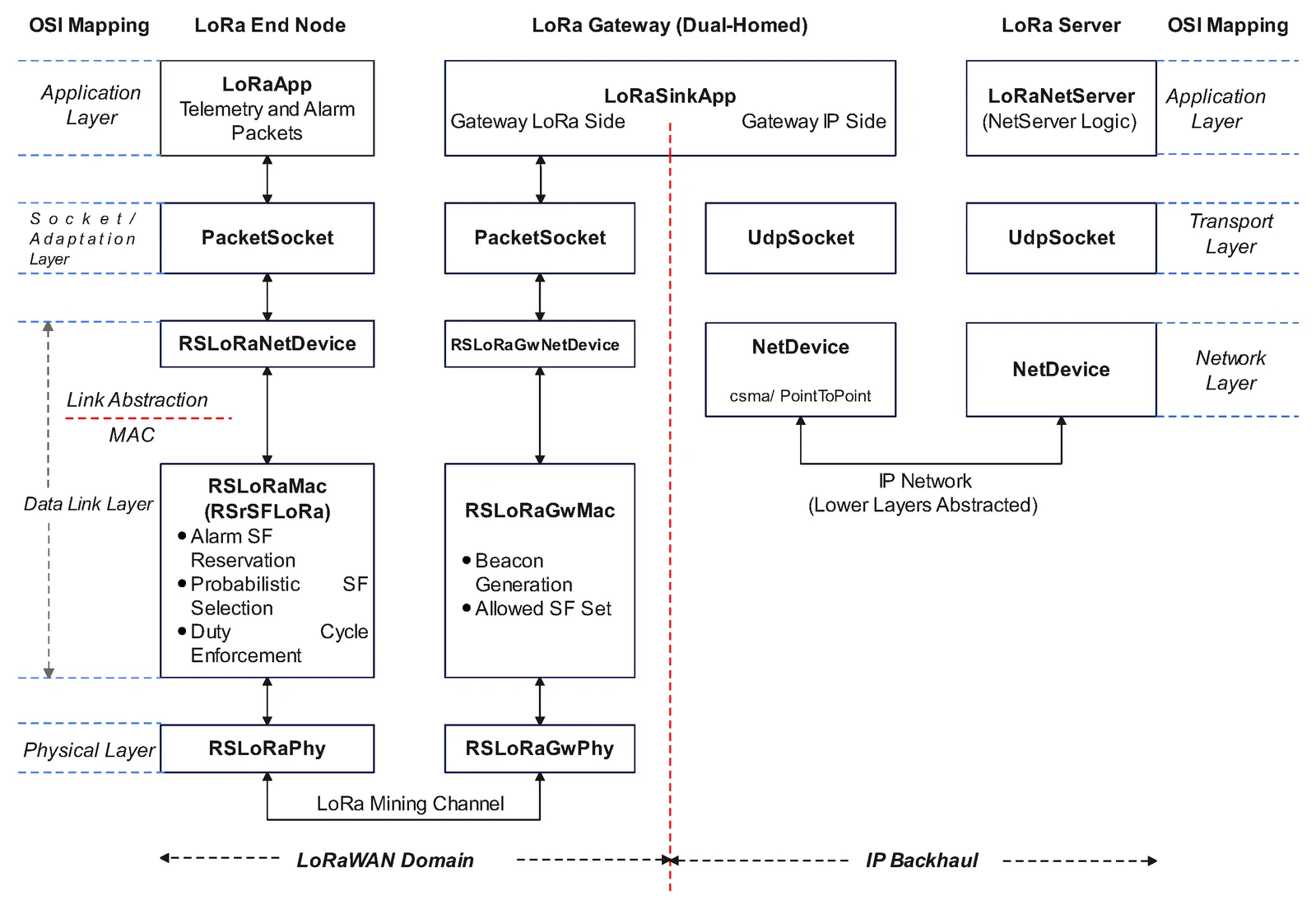
Implementation architecture of RSrSF-LoRa in ns-3.

This section provides a summary of important details on the implementation of our work in ns-3 and aligns it with a layered approach. It is worth noting that the LoRaWAN side (LoRa End Node to Gateway) of the implementation does not exactly fit into the common layered architecture such as the Open System Interconnection (OSI) or Transmission Control/ Internet Protocol (TCP/IP) model, while the IP side (Gateway to LoRa Server) does, with lower layers abstracted by the underlying IP network infrastructure. Figure 1 illustrates our implementation and mapping with layered architecture.

### 4.1. LoRaWAN Domain

The proposed RSrSF-LoRa system is implemented in ns-3 as a MAC-layer extension of RS-LoRa that reserves spreading factors (SFs) for alarm traffic while maintaining probabilistic allocation of SF for telemetry traffic. RSrSF-LoRa builds upon the existing RS-LoRa protocol by preserving its beacon synchronization and channel access procedures while introducing MAC-layer enhancements for spreading factor reservation and traffic-aware scheduling. These enhancements require corresponding support in both gateways and end devices; therefore, RSrSF-LoRa is intended as an extension of RS-LoRa rather than a fully interoperable replacement for unmodified deployments. The architecture maintains a clear separation between the application, link-layer control, and physical-layer radio modeling. For details on RS-LoRa implementation, refer to the study by Reynders et al. [6].

#### 4.1.1. Application Layer

At the application layer, LoRaApp and LoRaSinkApp are responsible for packet generation and semantic classification, reception, and processing. The end nodes generate periodic telemetry and event-driven alarm packets, annotated with metadata identifying their class. The gateway application is intentionally lightweight and receives LoRa packets, performs minimal classification, and forwards packets to the IP stack unchanged.

#### 4.1.2. Socket/Adaptation Layer

Although PacketSocket is sometimes informally associated with the transport layer, in ns-3, it serves as a link-layer socket interface that bypasses the network and transport layers, enabling direct communication between applications and NetDevices. This implies that communication between applications and the LoRa link layer is facilitated via PacketSocket, which provides a direct binding to link-layer NetDevice instances and bypasses the network and transport layers. Therefore, from an ns-3 perspective, the PacketSocket acts as a demultiplexing endpoint, as well as a mechanism to inject packets directly into RSLoRaNetDevice::Send(). This choice ensures that traffic differentiation remains visible to the MAC without distortion by transport-layer abstractions. This design reflects the non-IP nature of LoRa end devices and avoids unnecessary protocol overhead on the wireless link.

#### 4.1.3. Data Link Layer

The data link layer comprises both the MAC sublayer (RSLoRaMac and RSLoRaGwMac) and the link abstraction (RSLoRaNetDevice and RSLoRaGwNetDevice). RSrSF-LoRa implements SF reservation for alarms and probabilistic SF selection for telemetry entirely within the MAC sublayer, based on beacon information broadcast from the gateway. RSLoRaMac is the core of RSrSF-LoRa. ACK generation and retransmission logic is enabled only for alarm traffic, while telemetry traffic remains unacknowledged to minimize overhead. MAC entities also handle medium-access control, duty-cycle enforcement, and beacon processing, while the NetDevice exposes a standard interface to higher layers.

The gateway MAC (RSLoRaGwMac) mirrors RS-LoRa/RSrSF-LoRa logic symmetrically and is responsible for beacon generation, ACK handling, and MAC coordination. RSLoRaGwNetDevice aggregates PHY and MAC functionality and exposes received frames to the gateway application via a PacketSocket. This preserves symmetry with the end node architecture and simplifies packet forwarding.

#### 4.1.4. Physical Layer

The physical layer (RSLoRaPhy and RSLoRaGwPhy) models LoRa modulation, transmission power, propagation loss, and reception behavior. The end node PHY (RSLoRaPhy) implements the actual LoRa modulation and reception pipeline by applying the SF selected by the MAC, setting transmission power and timing, modeling interference, capture effect, and noise, and interfacing with the industrial (mining) channel model. The PHY is stateless with respect to traffic class; it executes MAC instructions without policy awareness. This confirms that RSrSF-LoRa is not a PHY-layer optimization.

The gateway PHY (RSLoRaGwPhy) performs multi-SF demodulation, Concurrent reception from multiple end nodes, and signal quality estimation. It feeds decoded frames upward to the gateway’s MAC and higher-layer processing, and subsequently to the network server, without imposing any traffic or scheduling policy. The gateway thus acts as a MAC coordinator (via beacons), reliability endpoint (via ACKs), and transparent bridge between LoRa and IP. RSrSF-LoRa logic remains strictly confined to the LoRa MAC.

### 4.2. IP Backhaul

The gateway is dual-homed: the LoRa interface participates in RSrSF-LoRa MAC and PHY operations with end nodes, while the IP interface communicates with the network server via UDP sockets. The network server operates exclusively at the application layer (LoRaNetServer). It receives packets via UDP/IP, processes telemetry data streams, triggers alerts or control logic for alarm packets, and optionally issues network-level control decisions.

## 5. Performance Evaluation and Discussion

**Figure 2:**
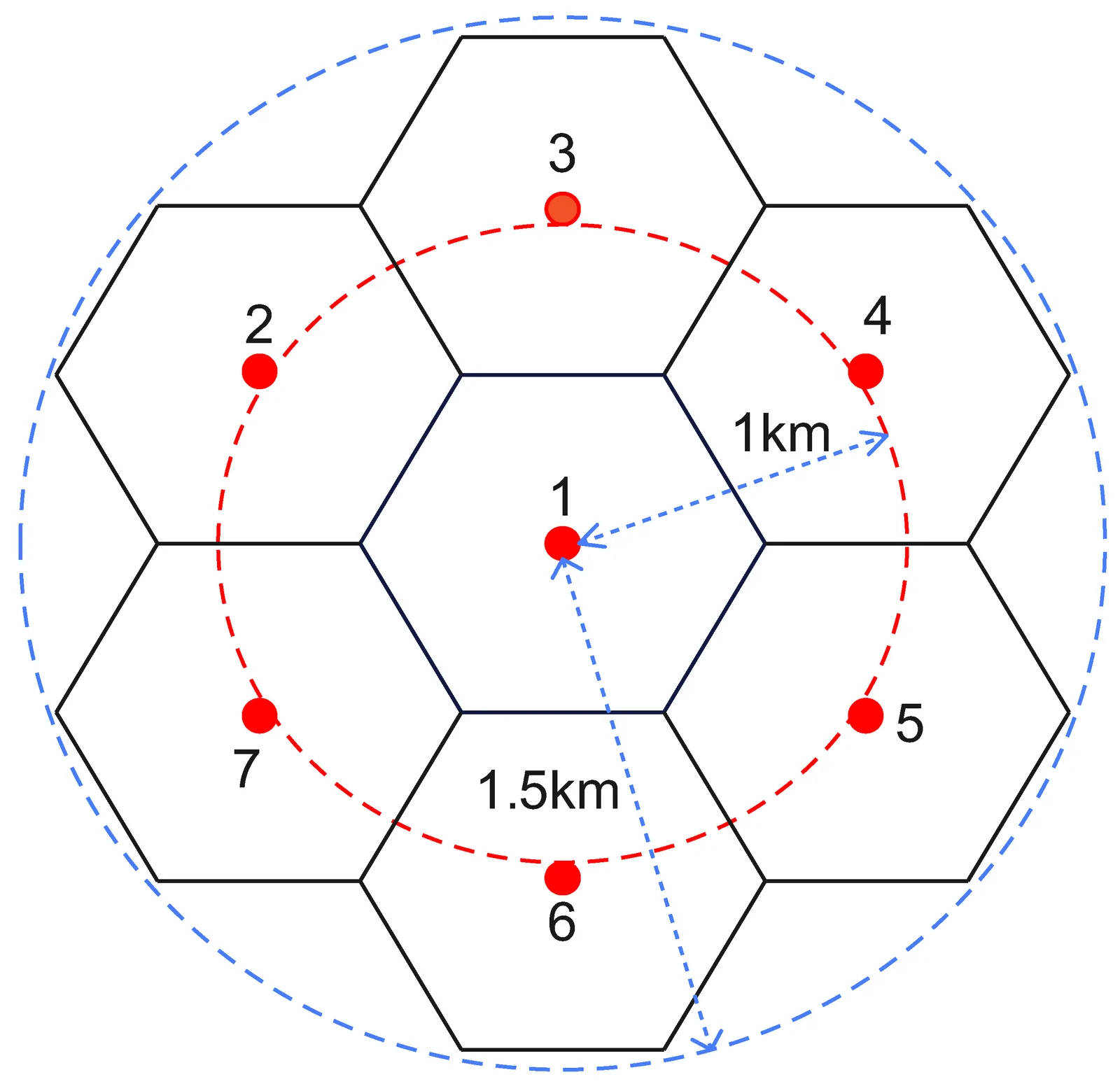
Gateway arrangement for the ns-3 implementation. The red dots and the corresponding numbers represent the gateway positions. Only the central gateway is used in the single-gateway setup.

We implemented a LoRaWAN network with LoRa-RSrSF running on the MAC layer using a single (1) gateway and a seven (7)-gateway arrangement. The gateways are 30 m above the ground, while the end nodes are 1 m above the ground. Taking the central gateway as a reference point, the nodes are distributed following the Poisson distribution within a radius of 1000 m for a single gateway and 1500 m for seven gateways, as shown in Figure 2. We assumed a mine in an urban setting where it is more likely to have small cells. In our implementation, we are using the EU 868 MHz band, spreading factors SF7 to SF12, and class A end nodes. The simulation parameters are tabulated in Table 4.

### 5.1. Single-Gateway Deployment Performance

#### 5.1.1. Reliability and Scalability Performance

**Figure 3:**
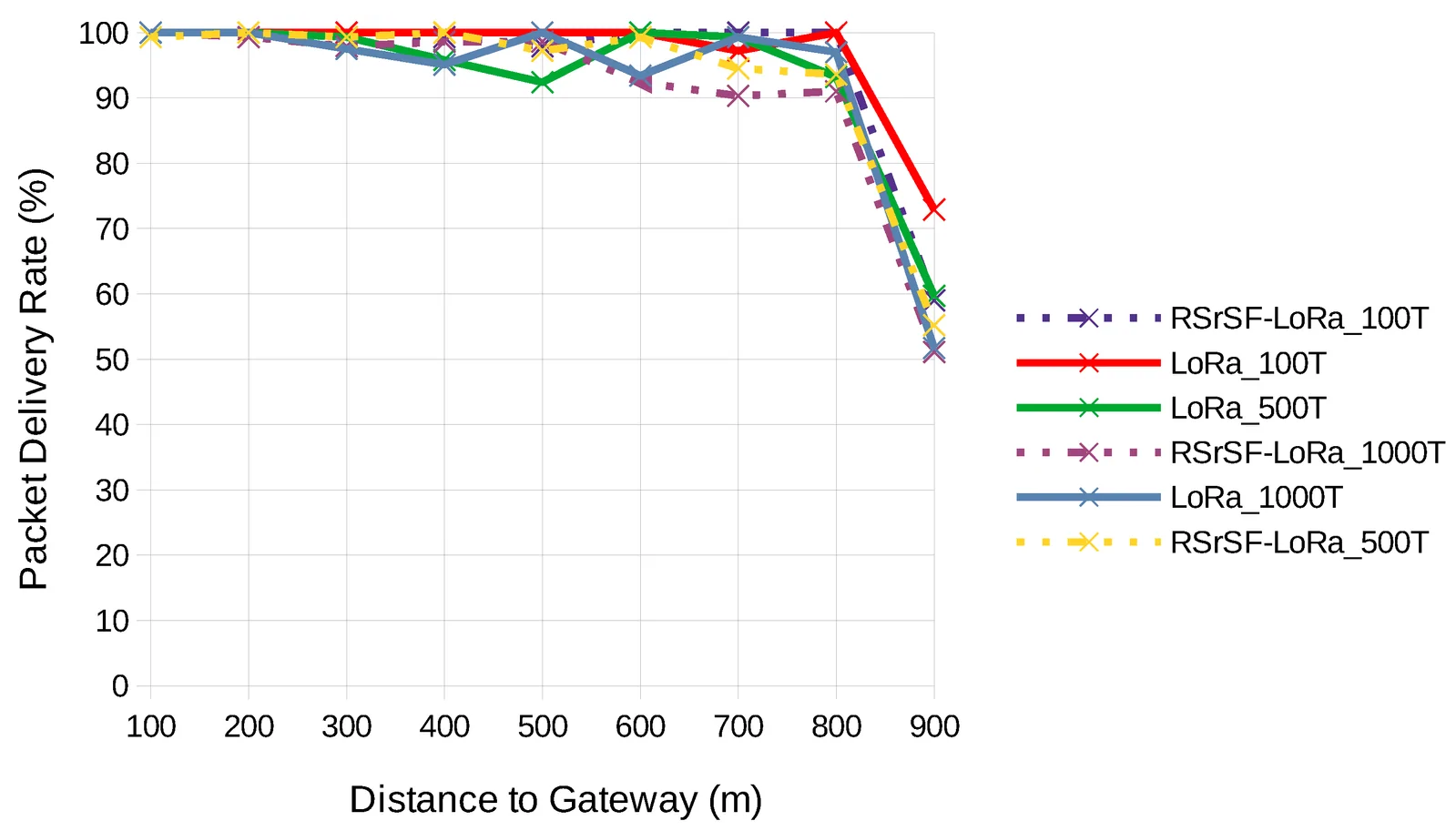
Telemetry node PDR versus distance for single-gateway scenario. RSrSF-LORa_NX denotes Mac layer protocol_Number of EDs and type of ED.

**Figure 4:**
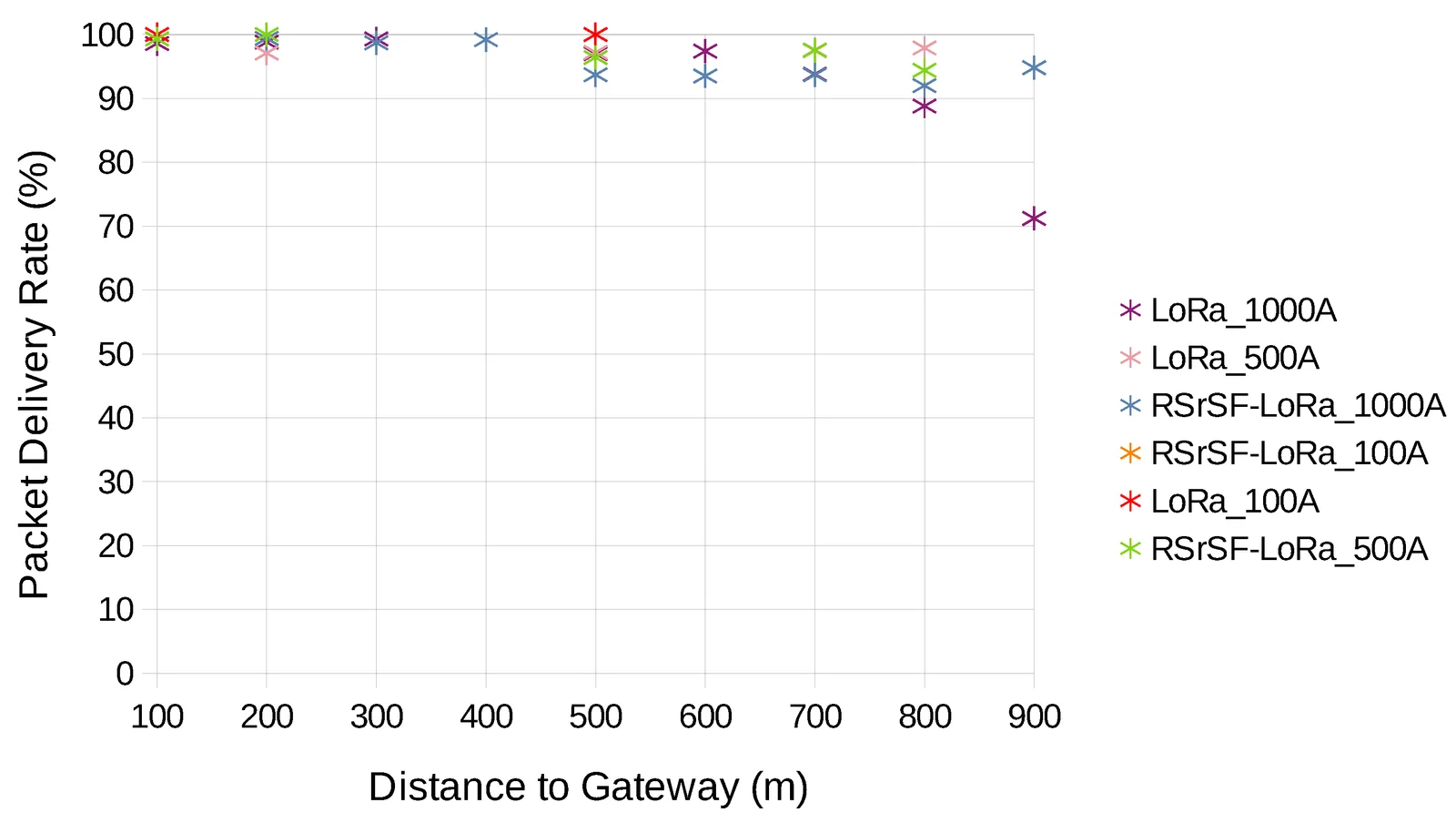
Alarm node PDR versus distance for single-gateway scenario. RSrSF-LORa_NX denotes Mac layer protocol_Number of EDs and type of ED.

The reliability and scalability performance of the proposed RSrSF-LoRa scheme was evaluated against conventional LoRaWAN under single-gateway deployments with varying network densities of 100, 500, and 1000 end nodes. In each scenario, 2% of the nodes generated priority alarm traffic, while the remaining nodes transmitted telemetry data. The packet delivery ratio (PDR) was used as the primary reliability metric and analyzed across communication distances of up to 1 km, representative of wide-area industrial monitoring environments. For reliable network communication, a PDR greater than 90% is required in mining environments. Figure 3 and Figure 4 show the PDR results for the single-gateway scenario for RSrSF-LoRa and LoRaWAN for telemetry and alarms, respectively.

For the 100-node scenario, both schemes achieved PDRs above 97% for telemetry reliability up to the range of 800 m, and it was predominantly 100% due to low channel contention. As the communication distance increased, performance degradation became noticeable, particularly at extreme ranges of 900 m and above, where combined effects of path loss, shadowing from metallic infrastructure, and reduced signal-to-noise ratio severely impact network reliability. Alarm transmissions remained highly reliable with PDRs of 100% for both schemes, confirming that low network load minimizes priority traffic impairment.

Under denser deployments of 500 nodes, contention effects became more pronounced. RSrSF-LoRa consistently maintained a high telemetry reliability of above 93% across most communication distances, with degradation occurring primarily at extreme ranges of 900 m and above. In contrast, conventional LoRaWAN showed greater variability at intermediate distances due to intensified channel competition and retransmission overhead. Alarm traffic analysis further highlighted the robustness of both schemes with PDR above 92%.

In the ultra-dense single-gateway scenario of 1000 nodes, congestion effects significantly influenced network performance. RSrSF-LoRa preserved high telemetry reliability across short ranges (0–300 m), with PDR above 97% and medium range (400–500 m) PDRs of above 98%, and maintained acceptable performance of PDR above 90% up to extended distances (600–800 m) before significant degradation occurred. Conventional LoRaWAN experienced some reliability fluctuations across distances, but PDRs were also above 90% up to 800 m. For priority alarm transmissions, RSrSF-LoRa maintained superior reliability consistency, with PDRs greater than 90% for all distances, while conventional LoRaWAN showed progressive degradation as transmission distance increased, with PDRs below 90% above 700 m. The results also indicate that RSrSF-LoRa is more stable in a dense single-gateway scenario because the drop in PDR values at 900 m when nodes are increased from 100 to 1000 is 8%, whereas for LoRaWAN it is 20%. These differences highlight the improved priority handling and transmission stability of the proposed mechanism.

Overall, the results confirm that RSrSF-LoRa provides better scalability and reliability consistency compared to conventional LoRaWAN in ultra-dense single-gateway deployments, particularly for priority alarm communication. The scheme mitigates congestion effects and maintains reliable telemetry and alarm communications, making it a possible option for large-scale industrial monitoring systems that require robust long-range performance. However, there is a limitation for both RSrSF-LoRa and LoRaWAN, such that we could not increase the number of nodes further than 1000 due to transmission degradation with distance, as shown in the PDR results, where 90% and above packet delivery success could only be achieved up to 800 m for both protocols. To verify the reliability of the results, the 95% confidence intervals (CIs) for telemetry PDR were determined for all distances considered and are shown in Table 5 and Table 6 for LoRaWAN and RSrSF-LoRa, respectively. Telemetry EDs represent 98% of the total number of EDs on the network, so the bulk of the traffic comes from telemetry transmission.

#### 5.1.2. Throughput Performance

**Figure 5:**
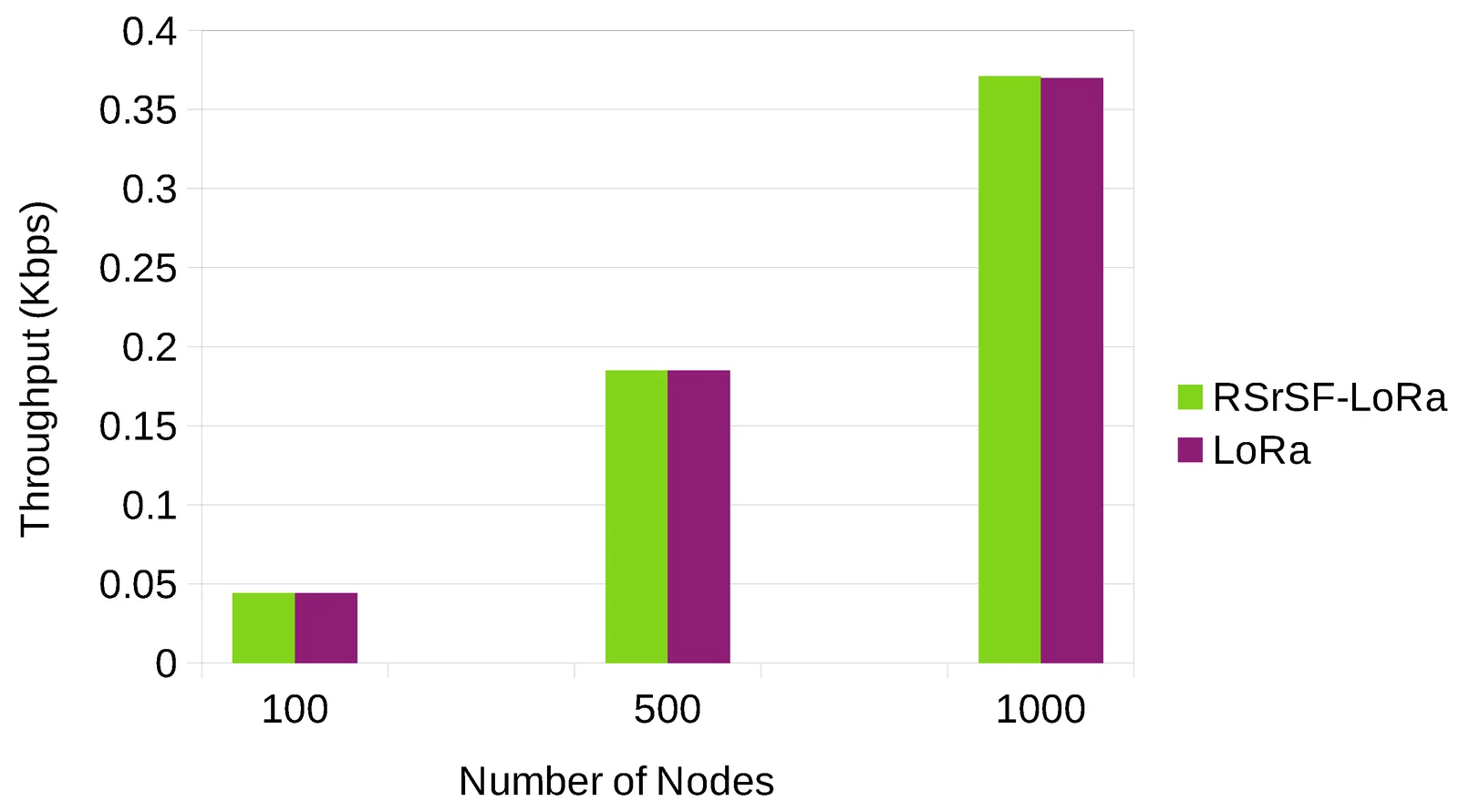
Throughput for single-gateway scenario.

Throughput reflects the efficiency of data transport under varying traffic loads and network densities. In LoRa-based systems, throughput is strongly influenced by spreading factor allocation, collision probability, and gateway diversity. Figure 5 shows the single-gateway throughput of RSrSF-LoRa and LoRaWAN.

The combined network throughput increases proportionally with node density, rising from 0.04 kbps at 100 nodes to 0.37 kbps at 1000 nodes in the single-gateway scenario. This near-linear scaling indicates efficient channel utilization and manageable collision levels as traffic load increases. The low absolute throughput values are consistent with the characteristics of LoRa networks, where small payload sizes and infrequent transmissions prioritise energy efficiency over high data rates. For the proposed RSrSF-LoRa scheme, the traffic model consists of periodic telemetry transmissions and Poisson-distributed alarm events, which together produce predictable long-term traffic behavior. This hybrid traffic structure contributes to stable throughput growth as node density increases.

#### 5.1.3. Fairness

Fairness performance was evaluated using Jain’s fairness index for both telemetry and alarm traffic. This is represented mathematically as

(13) $$J=\frac{{\left(\sum_{i=1}^{n}X_{i}\right)}^{2}}{n.\sum_{i=1}^{n}X_{i}^{2}}$$

where n represents the number of telemetry or alarm nodes for each network size being considered, i is each individual node, and $X_{i}$ represents the metric taken for each individual node.

**Figure 6:**
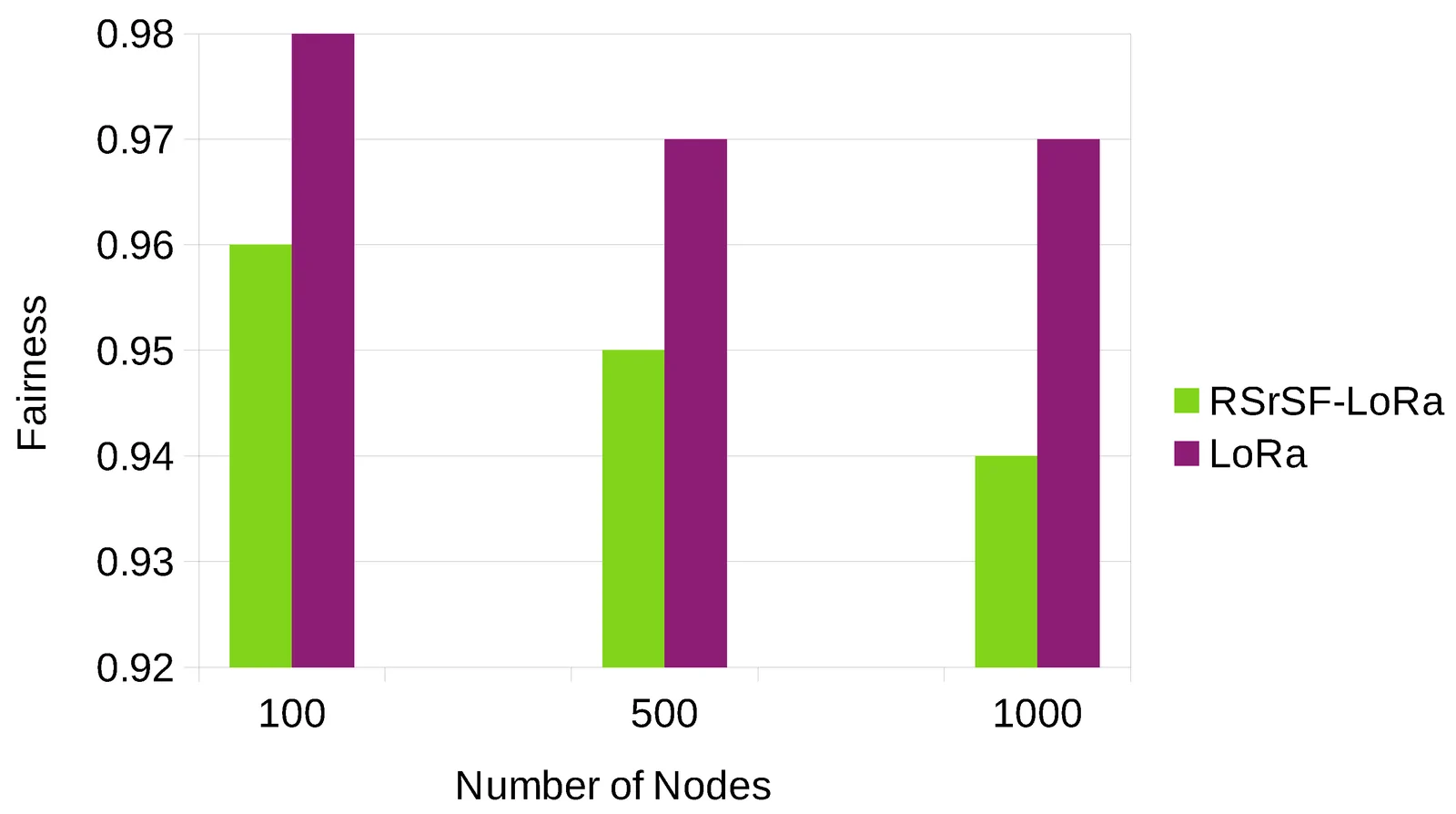
Fairness for telemetry transmission for single-gateway scenario.

**Figure 7:**
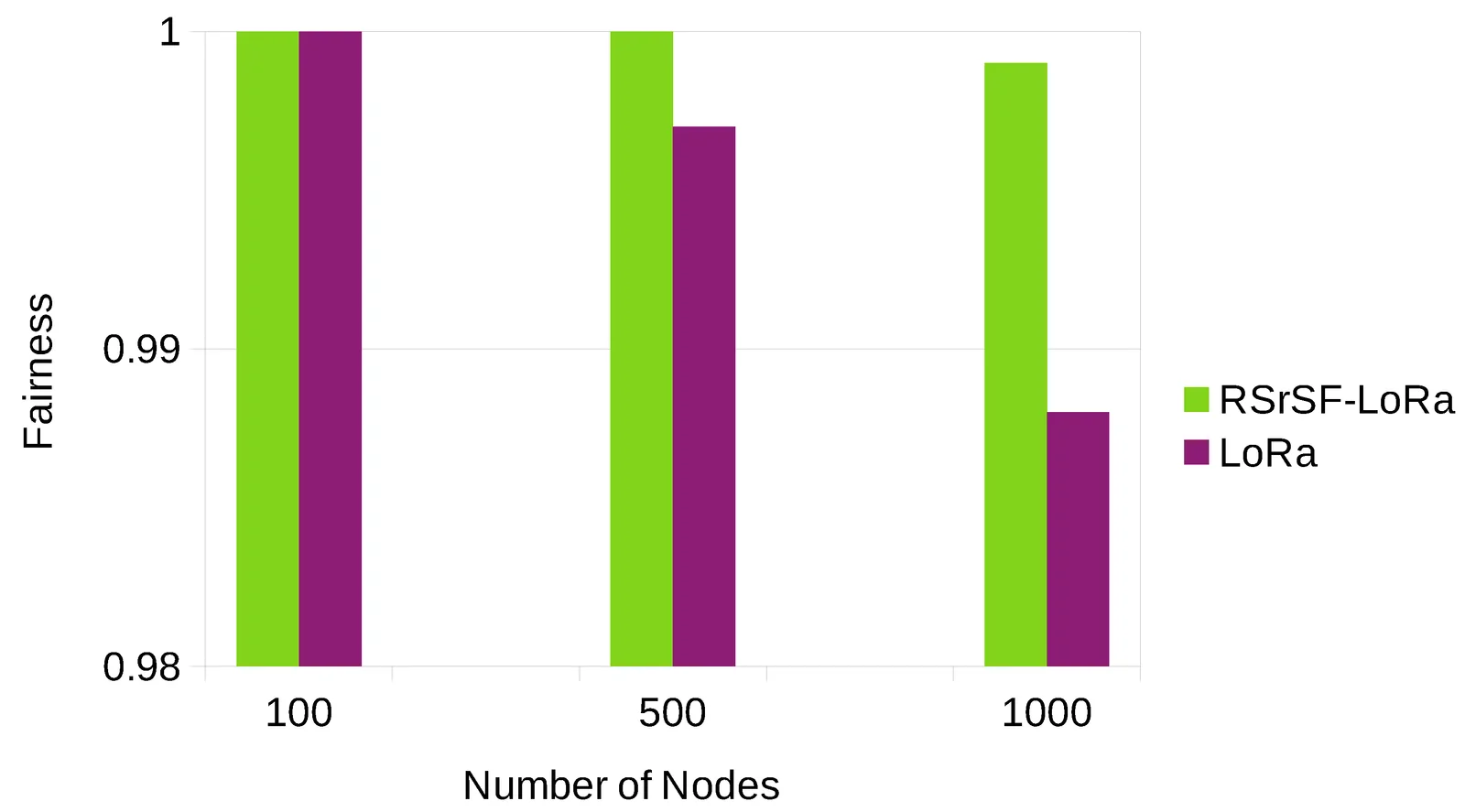
Fairness for alarm transmission for single-gateway scenario.

Figure 6 and Figure 7 show the results for fairness for RSrSF-LoRa and LoRaWAN for telemetry and alarm transmission, respectively. For telemetry nodes, RSrSF-LoRa achieves high fairness values ranging from 0.96 to 0.94 as node density increases, slightly lower than LoRaWAN, which maintains values between 0.98 and 0.97. This marginal reduction reflects the prioritisation of critical alarm traffic, resulting in controlled resource redistribution.

For alarm nodes, RSrSF-LoRa consistently achieves near-perfect fairness, maintaining values close to unity across all network sizes. In contrast, LoRaWAN exhibits a slight degradation in fairness at higher node densities, decreasing to 0.988 at 1000 nodes. This indicates the emergence of intra-class imbalance under increased contention. These results demonstrate that RSrSF-LoRa effectively ensures equitable access among critical nodes while maintaining high overall fairness, highlighting its suitability for reliability-sensitive applications in LoRa networks.

#### 5.1.4. Energy Consumption Performance

A normalized initial energy of 10 kJ is adopted, corresponding to the typical capacity of AA battery-powered LoRa end devices commonly used in general IoT deployments and a value that provides a realistic representation of practical node operation. The minimum and maximum energy in joules remaining after a simulation period of 24 h are tabulated below (Table 7) for alarms and telemetry.

Across all scenarios, both schemes exhibit minimal absolute energy depletion after 24 h due to the low-duty-cycle nature of LoRa communications, with total energy consumption remaining below 3 J per node. As the number of nodes increases, a gradual increase in energy consumption is observed for both schemes, mainly due to increased channel contention, collision probability, and retransmission activity. LoRaWAN consistently demonstrates low average energy consumption in all node densities. In contrast, RSrSF-LoRa exhibits a controlled increase in energy consumption with network size, accompanied by a consistently balanced energy distribution among nodes. The key observations are that alarm nodes have a higher energy consumption (down to ≈9997.23 J at 1000 nodes), while telemetry nodes have a tight and stable range (≈9999.04–9999.32 J in all cases). The increased energy consumption observed for the alarm nodes is a direct consequence of the scheme’s prioritization mechanism, ensuring reliable delivery of critical traffic even under high network load.

Although RSrSF-LoRa incurs slightly higher energy consumption compared to LoRaWAN, particularly for alarm nodes, this increase remains marginal (≤3 J over 24 h) relative to the available 10 kJ energy budget. This marginal energy overhead is justified by the improvements in transmission reliability, fairness, and network stability achieved by the proposed scheme. Overall, the results demonstrate that RSrSF-LoRa provides a scalable and energy-efficient solution for dense LoRa networks by maintaining balanced energy consumption and reliable communication. It should also be noted that the increased energy usage observed in RSrSF-LoRa for alarm nodes does not significantly reduce the practical lifetime, as it remains within the expected operational bounds of LoRa-based systems. These findings confirm that the proposed RSrSF-LoRa scheme achieves improved reliability and fairness without compromising the practical lifetime constraints of LoRa end devices.

### 5.2. Multi-Gateway Deployment Performance

#### 5.2.1. Reliability and Scalability Performance

**Figure 8:**
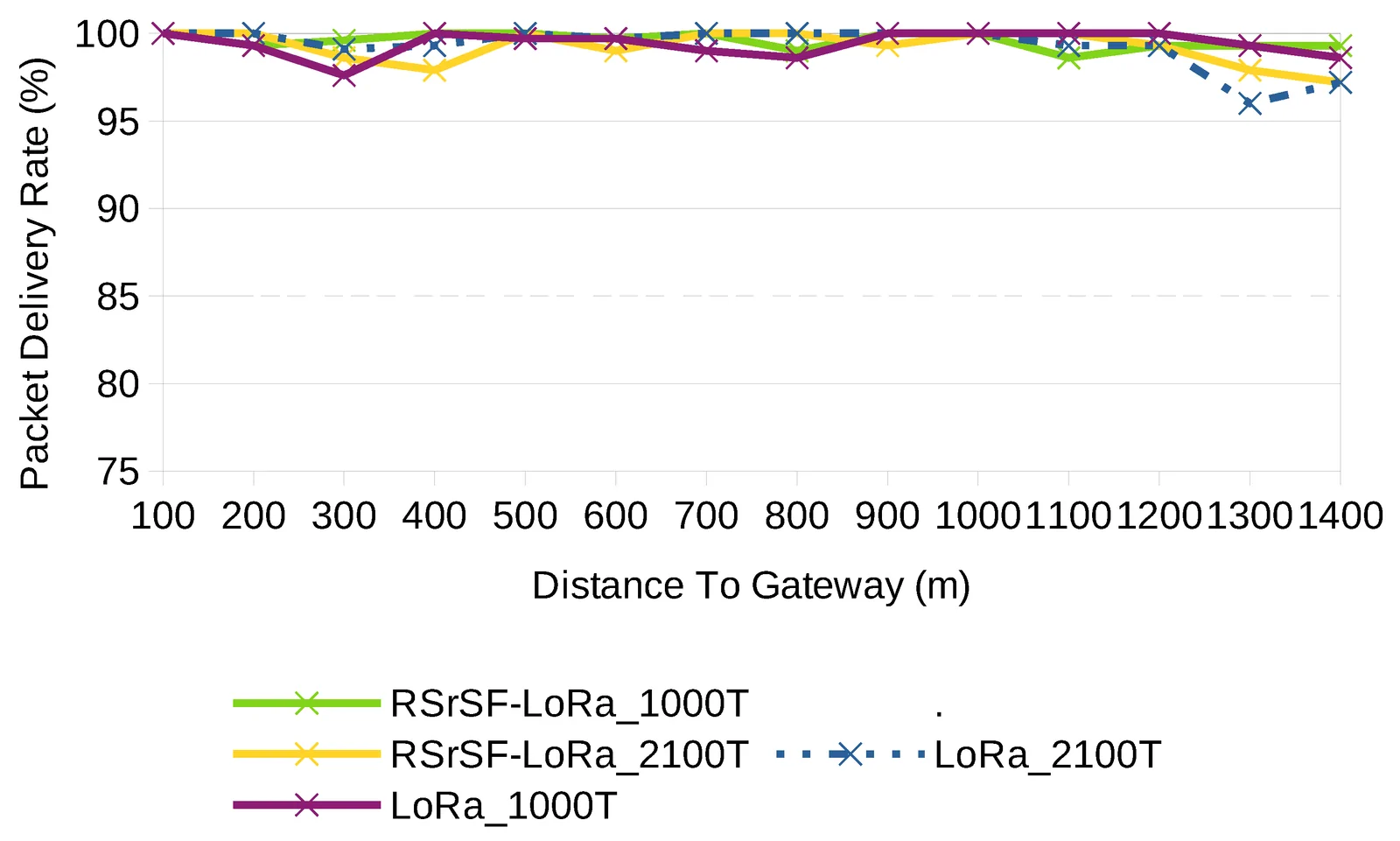
Telemetry node PDR versus distance for seven-gateway scenario. RSrSF-LORa_NX denotes Mac layer protocol_Number of EDs and type of ED.

**Figure 9:**
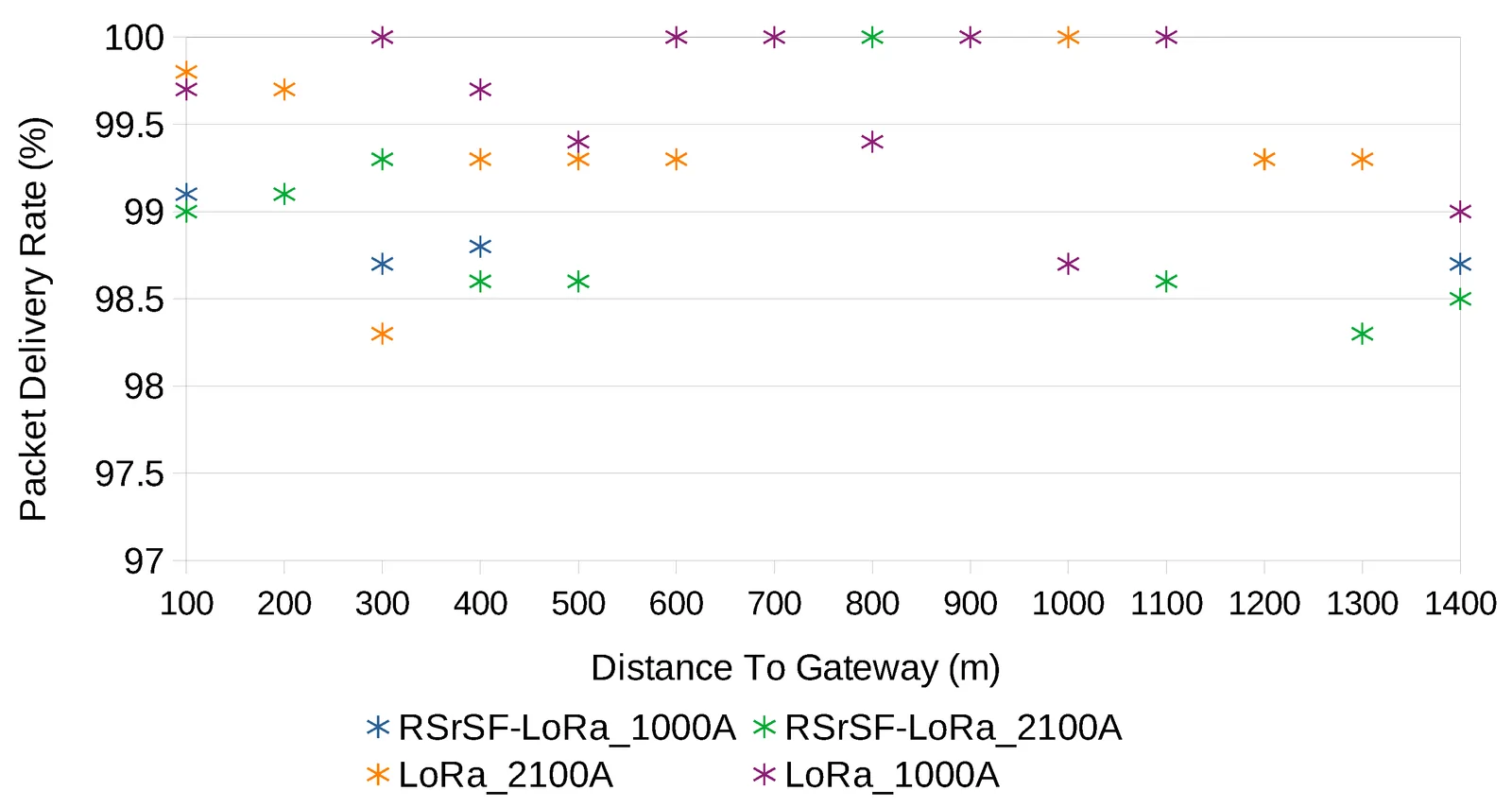
Alarm node PDR versus distance for seven-gateway scenario. RSrSF-LORa_NX denotes Mac layer protocol_Number of EDs and type of ED.

To assess the impact of infrastructure densification, performance was further evaluated using seven-gateway deployments for large-scale networks comprising 1000 and 2100 nodes. Among these devices, 2% generated priority alarm traffic, while the remaining nodes transmitted telemetry data. The PDR was analyzed over extended communication distances ranging from 100 m to 1.5 km to assess reliability and scalability under wide-area industrial monitoring conditions. Gateway diversity improves spatial reception, balances traffic load, and reduces the probability of packet collision, thus enhancing the overall robustness of the network. Figure 8 and Figure 9 show the PDR results for the seven-gateway scenario for RSrSF-LoRa and LoRaWAN for telemetry and alarms, respectively.

For the 1000-node multi-gateway scenario, both schemes achieved near-perfect telemetry reliability across the evaluated coverage range with PDRs above 97%. RSrSF-LoRa maintained highly stable performance with minimal variation, demonstrating effective utilization of gateway diversity to mitigate interference and propagation losses. Conventional LoRaWAN also benefited from load distribution and reception diversity but exhibited slightly greater performance variability at several intermediate distances due to residual contention effects. Alarm transmissions for both schemes achieved excellent reliability, with RSrSF-LoRa providing marginally stronger consistency.

When the node density was increased to 2100 nodes, multi-gateway deployment significantly alleviated congestion effects. RSrSF-LoRa sustained good telemetry reliability across almost all communication distances despite doubling the traffic density, with PDR above 97% at most distances. Conventional LoRaWAN also maintained high reliability with very minimal reductions at certain long-range distances, indicating sensitivity to intensified channel competition. Alarm traffic remained highly reliable for both schemes, although RSrSF-LoRa demonstrated slightly more uniform performance.

These findings confirm that infrastructure densification substantially improves network stability and scalability. When combined with the proposed RSrSF-LoRa mechanism, multi-gateway architectures enable reliable communication across extended coverage areas and increased node densities. The approach effectively maintains stable telemetry and priority traffic performance in extended coverage areas, demonstrating strong suitability for large-scale monitoring applications. In addition, the scheme can distribute SFs after making a reservation for alarms without affecting telemetry transmission for both deployment options. Table 8 and Table 9 show the 95% confidence intervals for PDR results for the multiple-gateway scenario for both LoRaWAN and RSrSF-LoRa.

#### 5.2.2. Throughput Performance

**Figure 10:**
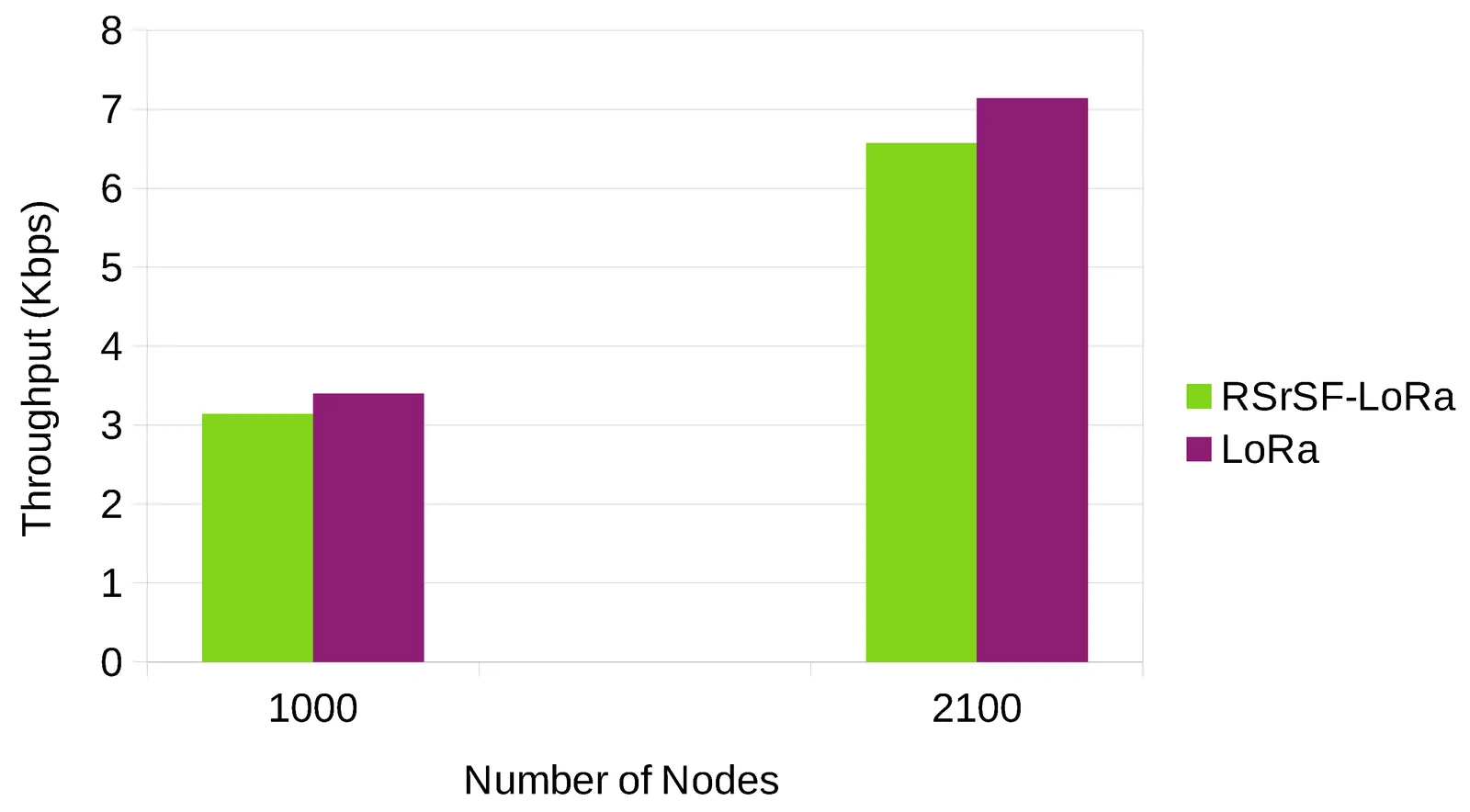
Throughput for seven-gateway scenario.

In the seven-gateway scenario, both schemes exhibit substantial throughput improvement due to enhanced spatial diversity and reduced collision probability. Throughput increases with node density for both protocols, confirming scalable traffic handling. Figure 10 shows the throughput for RSrSF-LoRa versus LoRaWAN. LoRaWAN achieves slightly higher aggregate throughput, exceeding RSrSF-LoRa by 0.55 kbps and 0.57 kbps at 1000 and 2100 nodes, respectively. This marginal advantage suggests that LoRaWAN prioritizes raw channel utilization, while RSrSF-LoRa introduces traffic management mechanisms that may slightly reduce aggregate throughput.

The results demonstrate that RSrSF-LoRa maintains competitive throughput performance under dense large-scale deployments while enabling enhanced quality-of-service provisioning. However, for the seven-gateway scenario, it was observed that approximately 8–8.6% (higher value for 2100 nodes) of RSrSF-LoRa nodes did not transmit any packets during the simulation period. Among these were two–three alarm nodes (with higher values for 2100 nodes), which is critical for delay-sensitive communication. This behavior suggests possible scheduling starvation or channel access imbalance under high network density, potentially limiting full network utilization.

#### 5.2.3. Fairness

**Figure 11:**
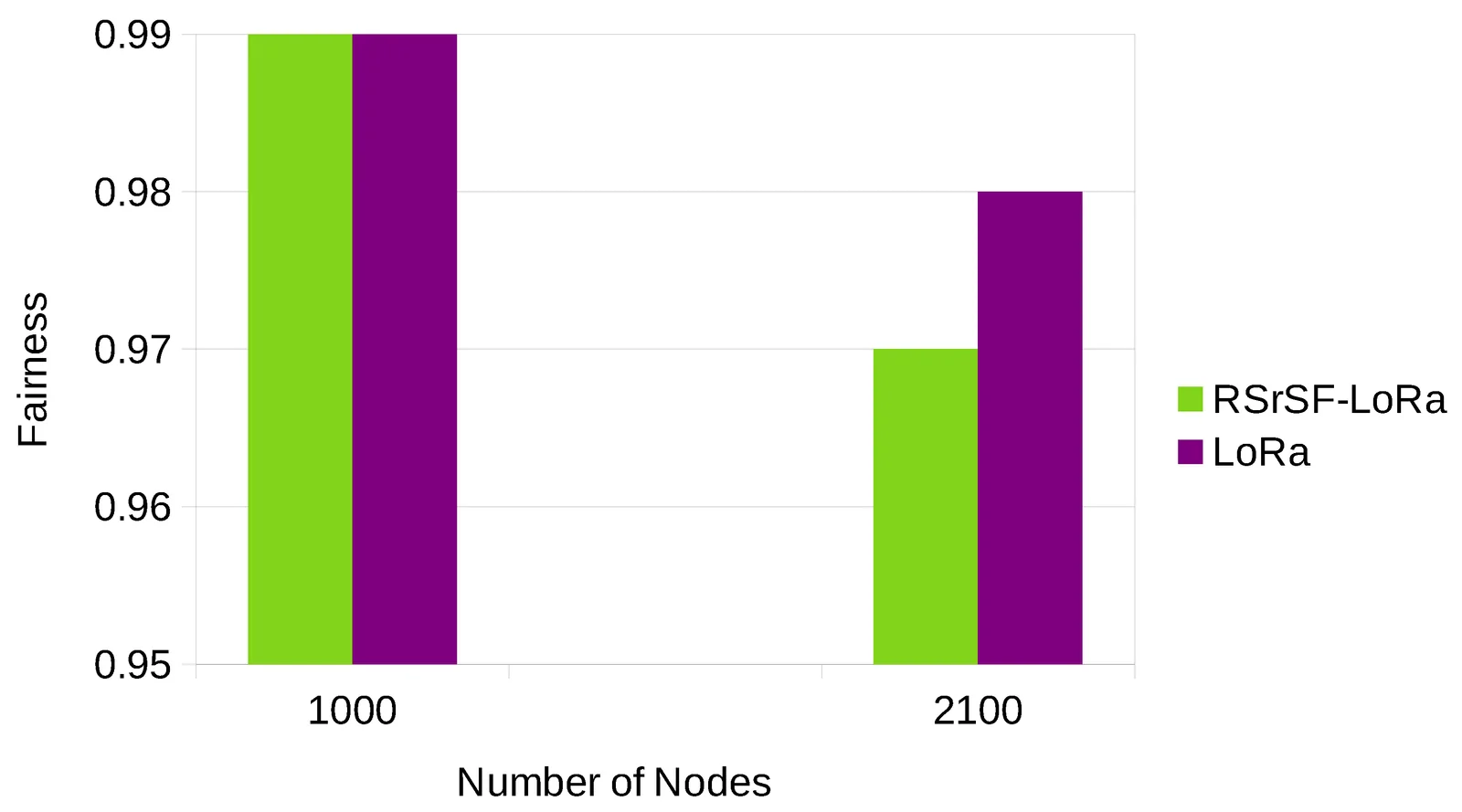
Fairness for telemetry transmission for seven-gateway scenario.

**Figure 12:**
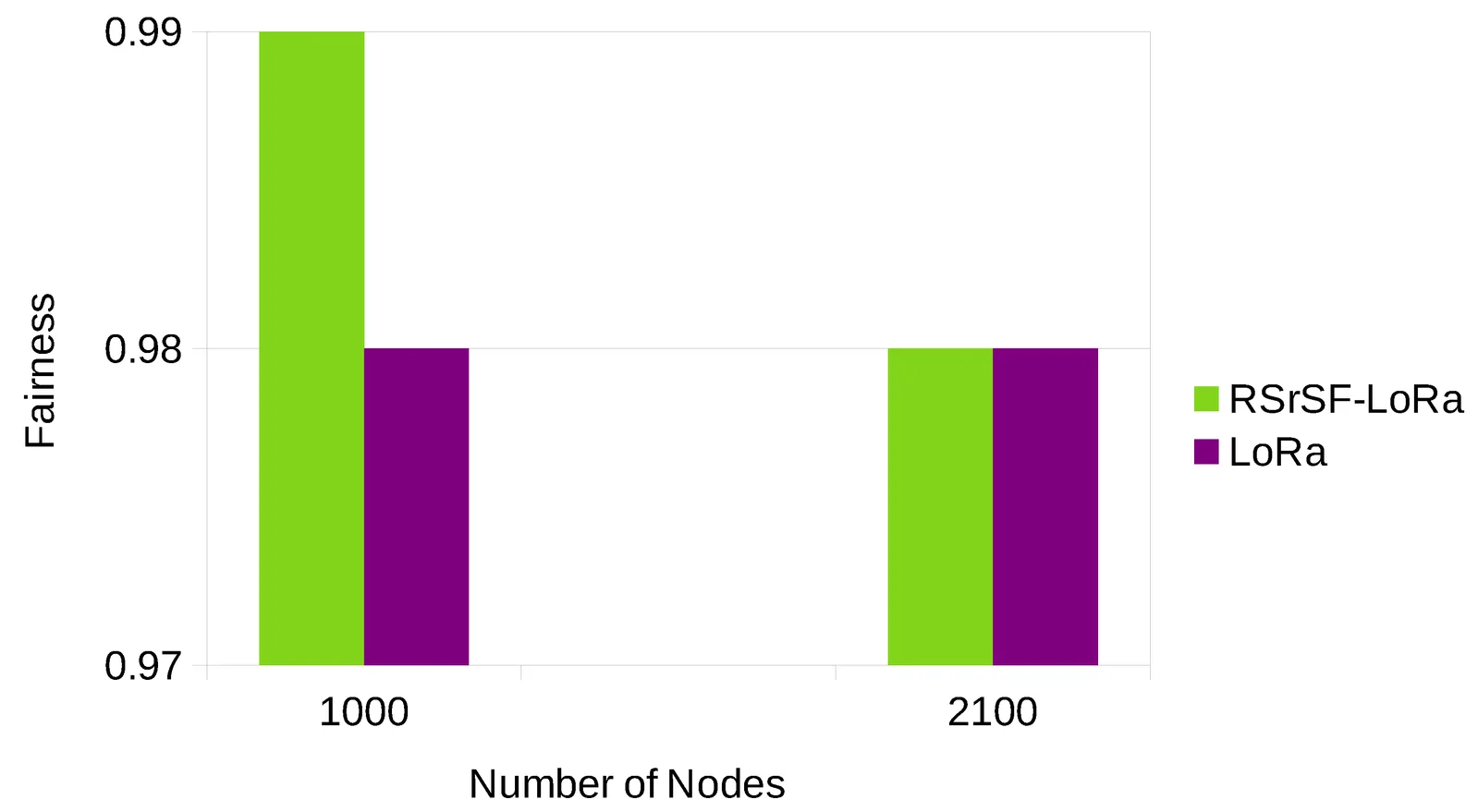
Fairness for alarm transmission for seven-gateway scenario.

In the seven-gateway scenario, both RSrSF-LoRa and LoRaWAN achieve high levels of fairness for telemetry and alarm traffic, with Jain’s fairness index exceeding 0.97 for all evaluated network sizes. The use of multiple gateways improves spatial reuse and reduces contention, resulting in a more uniform resource distribution. Figure 11 and Figure 12 show the results obtained for fairness in both schemes for telemetry and alarm transmission, respectively.

For telemetry traffic, both schemes exhibit comparable fairness, with values of 0.99 at 1000 nodes and slight reductions at 2100 nodes due to increased contention. Similarly, alarm traffic maintains near-uniform fairness across both schemes, indicating balanced resource allocation among transmitting nodes. However, as already highlighted, a small fraction of nodes, approximately 8–8.6% of the nodes in the RSrSF-LoRa scheme, did not transmit during the simulation period, including two–three alarm nodes. This behavior is not observed in LoRaWAN and suggests the presence of transmission starvation under high-density conditions. As Jain’s fairness index is calculated on active transmitting nodes, this metric does not capture disparities in channel access. These findings highlight that while RSrSF-LoRa ensures high fairness among transmitting nodes, additional mechanisms are required to guaranty transmission opportunities for all nodes, particularly critical alarm traffic in dense LoRa network deployments.

#### 5.2.4. Energy Consumption Performance

Similar to the single-gateway network, a normalized initial energy of 10 kJ is assumed for all end devices. Across both node densities, the results indicate that the overall energy consumption remains minimal, with the total energy depletion per node remaining below approximately 2.5 J after 24 h, reflecting the low-duty-cycle nature of LoRa communications. Table 10 shows the minimum and maximum energy remaining after a simulation period of 24 h for both alarms and telemetry.

The introduction of multiple gateways significantly improves energy efficiency as it enables the use of lower spreading factors, thus reducing time-on-air and transmission energy. Consequently, both LoRaWAN and RSrSF-LoRa benefit from reduced retransmission overhead and improved link reliability compared to the single-gateway scenario.

LoRaWAN consistently demonstrates low average energy consumption for both network sizes. For the alarm nodes, energy levels remain tightly clustered, indicating minimal retransmission activity. However, telemetry nodes exhibit noticeable variability in energy levels, with values ranging from approximately 9998.8 J to 9999.5 J. In contrast, RSrSF-LoRa exhibits a controlled and predictable energy consumption pattern across both network sizes. Alarm nodes consistently show higher energy consumption compared to LoRaWAN, with minimum energy levels reaching approximately 9997.66 J at 2100 nodes. This increase is attributed to the priority mechanism of the scheme, which ensures the reliable delivery of critical traffic. For telemetry nodes, RSrSF-LoRa maintains a tightly bounded energy distribution, with values remaining within a narrow range (approximately 9999.02 J to 9999.84 J). This indicates balanced channel access and effective resource allocation, even as the network scales from 1000 to 2100 nodes.

Although RSrSF-LoRa incurs a marginal increase in energy consumption, particularly for alarm nodes, this overhead remains negligible relative to the available 10 kJ energy budget. Importantly, the resulting energy consumption levels remain well within the practical lifetime constraints of typical LoRa end devices, which are primarily limited by battery chemistry rather than energy usage. Overall, the results demonstrate that multi-gateway deployment enhances energy efficiency for both schemes.

### 5.3. Robustness Considerations and Limitations

This section summarises the impact of beacon loss and synchronisation errors and the limitations of our work.

**Impact of Beacon Loss and Synchronization Errors:** RSrSF-LoRa inherits the beacon-based synchronization mechanism of RS-LoRa, whereby end devices periodically receive gateway beacons to maintain a common time reference for channel access and spreading factor selection. When a beacon is missed, a node continues operating using its most recently acquired synchronization information. Because oscillator drift over a single-beacon interval is generally small, occasional beacon losses are expected to have only a minor impact on scheduling accuracy. However, repeated beacon losses may gradually increase synchronization error, causing telemetry nodes to incorrectly estimate reserved spreading factor availability or alarm nodes to transmit using outdated reservation information. Such synchronization errors may increase packet collisions, transmission latency, and packet loss. Nevertheless, because RSrSF-LoRa reserves spreading factors rather than enforcing strict time-slot scheduling, its synchronization requirements are less stringent than those of conventional TDMA-based protocols. The current study assumes reliable beacon dissemination provided by the underlying RS-LoRa protocol and does not explicitly evaluate beacon loss or clock drift. Investigating the robustness of RSrSF-LoRa under realistic synchronization impairments constitutes an important direction for future work.
**Intelligent Coordination:** Although conceptually, RSrSF-LoRa has been implemented, it does not include intelligent coordination to determine the highest SF for the alarms without adding any delay.
**Transmission Failure:** For the seven-gateway scenario, it was observed that approximately 8–8.6% of RSrSF-LoRa nodes did not transmit any packets during the simulation period. Among these were two–three alarm nodes, which is critical for delay-sensitive communication. This behavior suggests possible scheduling starvation or channel access imbalance under high network density, potentially limiting full network utilization.

## 6. Conclusions

The simulation results indicate that both RSrSF-LoRa and LoRaWAN achieve high reliability in multi-gateway deployments, with packet delivery ratio (PDR) values exceeding 97%. However, in single-gateway dense scenarios with 1000 nodes, RSrSF-LoRa significantly improves the reliability of alarm messages, extending the acceptable PDR range from 700 m (LoRaWAN) to 900 m. Throughput and fairness remain comparable across both mechanisms, while energy consumption for alarm nodes in RSrSF-LoRa is slightly higher due to reserved transmission, but overall energy usage remains acceptable. These results demonstrate that RSrSF-LoRa provides reliability benefits in high-density industrial deployments without compromising the overall performance of the network, providing valuable guidance for designing reliable and energy-efficient IIoT networks in smelter environments.

Therefore, our proposals for future work are as follows: for the single-gateway network, there is a need to determine how to position the gateway in such a way that we can incorporate a relay device (repeater) that would increase the range with minimal cost in infrastructure compared to the seven-gateway arrangement. A limitation observed in this study for dense multi-gateway deployments is that a small fraction of nodes experienced transmission starvation. Future enhancements will incorporate starvation-avoidance and guaranteed-access mechanisms for critical alarm traffic to further enhance reliability in dense multi-gateway deployments. Although conceptually, RSrSF-LoRa has been implemented, further studies are needed to enhance the work and make it possible to apply RSrSF-LoRa in real-world applications by incorporating intelligent coordination in determining the highest SF for the alarms without adding any delay. This study compared RSrSF-LoRa with legacy LoRaWAN; however, future work should also include a comparison with the more recent LoRa MAC enhancement schemes. It would also be worthwhile to investigate and resolve the challenges of alternative spreading factor allocation strategies to see if they will produce a better result, and observed performance improvements should be explained using collision statistics. This should also include evaluation of average and tail latency, deadline-miss probability, number of retransmissions, and alarm-delivery outage probability. A further study will investigate incorporation of Forward Error Correction such as the 4/8 Hamming code without disturbing the required low bit rate transmission for LoRaWAN, as well as integrating Finite Impulse Response (FIR) or Infinite Impulse Response (IIR) filters after demodulation at the receiver to increase immunity to the propagation effects of the mining channel. Another study will exploit the concept of First-Slotted Carrier-Sense Multiple Access with Collision Avoidance (LFS-CSMA) by prioritising alarm messages, transmitting them earlier within a given time slot, and then telemetry messages will check the channel availability before sending and avoid collisions if they detect an ongoing transmission.

This study has also demonstrated the potential of LoRaWAN to improve air-quality monitoring for poisonous gases resulting from copper smelting. Some traditional wireless technologies fail at the smelter within a range of 600 m. In addition, current systems use sensors that depend on grid-connected power, making it difficult to deploy many sensor nodes since they are limited to deployment in only a few strategic locations or facilities. LoRa end nodes are battery-powered; hence, they are feasible for dense deployments.

## Figures and Tables

**Table 1:** Comparison summary of related works and presented work.

Paper/Year	Application Area	Proposed Protocol/Strategy	Purpose of Work	Obtained Result	Implementation
[8]/2020	Underground mine	Redundant retransmission aided adaptive latency reduction	Adjusting ACK-timeout based on air time of previous uplink transmission and contention stage	Better performance compared with LoRaWAN on average time delay for emergency traffic (alarms)	Simulation using LoRaSim
[9]/2023	Harsh underground environments (mining and tunneling industry)	Single-channel MAC protocol based on TDMA and algorithm to support roaming	Managing slot assignments to eliminate collisions and minimise the number of slots for a given number of gateways	The MAC protocol provides efficient unicast services for data transmission and collection, improving the performance of LoRa networks	Development of firmware based on the MAC protocol and MATLAB used for algorithm development
[10]/2022	Underground gold mine	Foundation models for LoRa signal behavior in block cave mining	Collecting mining measurements of LoRa radio propagation in drift segment of underground gold mine for LOS and NLOS communication for developing foundation models for LoRa signal behavior and determining the effect of SF on signal strength	1. For LOS communication, path loss is modeled using the path loss law with a 1.25 path loss index, and variability in signal strength using a log-normal distribution with mean 4.5. 2. Short-distance NLOS communication is modeled using Fresnel diffraction with path loss = 2 and free space model. 3. SF has a variable impact on RSSI and a consistent effect on SNR.	Measurements taken on site in a block cave gold mine
[11]/2024	Underground coal mine	Data rate algorithm for mesh network topology	Optimising data rate and maximising coverage distance with minimal repeaters	1. Implementation of algorithm achieves 1.46 Kbps data rate at SF9, bandwidth 125 KHz and transmission power 11 dBm and extends battery life. 2. Deployed end nodes achieve a maximum distance of 180 m.	Deployment of prototype and testing in underground coal mine
[7]/2020	Indoor and open-field industrial environment	Spreading factor allocation strategies for alarms and telemetry messages	Prioritising alarms without impairing telemetry	high reliability with reasonable delay of alarm messages	Simulation using ns-3
[12]/2021	Industrial environment	-	Analysis of higher-layer impact of PHY-layer models using industrial channel models for confirmed/unconfirmed traffic and multi-gateway deployment utilising EU 2.4 GHz frequency	Utilising 2.4 GHz in LoRaWAN network improved transmission time by 80% decreasing from 75 ms to 14 ms and PDR above 90% was achieved due to extended bandwidth	Simulation using ns-3
[6]/2018	Urban environment	RS-LoRa Mac Layer	Improve reliability and scalability of LoRaWAN	In a single-gateway network of 1000 nodes, RS-LoRa reduced the packet error ratio of legacy LoRaWAN by 20%.	Simulation using ns-3
[13]/2022	General	Longest First-Slotted Carrier-Sense Multiple Access with Collision Avoidance	Combine slotted Aloha and CSMA schemes so that longer frames are transmitted earlier within a given time, and shorter frames check the channel availability before sending to avoid collisions	Far fewer collisions than traditional MAC mechanisms, thus improving scalability of LoRaWAN significantly	Analytical modelling and numerical analysis
Presented work	Mining smelter environment	Hybrid MAC mechanism RSrSF-LoRa	Reliable transmission of pollution-monitoring traffic by prioritising alarms through SF reservation and distributing remaining SFs among telemetry end nodes to prevent overcrowding in an inappropriate SF	In a dense single-gateway scenario with 1000 nodes, RSrSF-LoRa improves alarm message reliability, extending acceptable PDR range from 700 to 900 m in comparison with LoRaWAN	Simulation using ns-3

**Table 2:** Elementary operation counts for Algorithm 1 with |I|=3 and |S|=6.

Step	Lines	Operations
*Alarm end node*		
Initialisation	2–4	3
Channel search loop	5–11	15
Channel-found test	12	1
Weighted SF draw, Equation (4)	13	37
rSF confirmation loop ^a^	17–22	6
Transmit power computation	24	5
Time-on-air and random offset	30–31	10
**Total**		**77**
*Telemetry end node*		
Initialisation	35–36	2
Channel search loop	38–44	15
Channel-found test	45	1
Weighted SF draw, Equation (4)	46	37
rSF collision test ^a^	47	2
Resampling on collision ^a,b^	48	43
Transmit power computation	50	5
Time-on-air and random offset	56–57	10
**Total (best/worst case)**		72/115

^a^ Step introduced by RSrSF-LoRa; all remaining steps are inherited from RS-LoRa. ^b^ Executed only when the drawn SF coincides with the reserved SF and rSF<12.

**Table 3:** Complexity comparison of RSrSF-LoRa with legacy LoRaWAN and alternative reliability mechanisms.

Mechanism	ED Time	ED State	GW Time	Coordination	Extra RX
Legacy LoRaWAN	Θ(1)	Θ(1)	Θ(1)	None	No
LFS-CSMA [13]	Θ(1)	Θ(1)	Θ(1)	None	Yes ^a^
TDMA MAC [9]	Θ(1)	Θ(N)	Θ(N)	Slot assignment	No
RS-LoRa [6]	Θ(|I|+|S|)	Θ(|I||S|)	Θ(|I||S|)	Beacon	No
RSrSF-LoRa	Θ(|I|+|S|)	Θ(|I||S|)	$\Theta \left(\left|I\right|\left|S\right|\right)+N_{a}$	Beacon	No

^a^ Carrier sensing requires the receiver to be active before each transmission, incurring an energy cost not present in scheduled mechanisms.

**Table 4:** Summary of simulation parameters.

Parameter	Setting	Unit
Number of nodes	100 to 2100	−
Maximum distance to Gateway 1	1000 or 1500	meter
Distance between gateways	1000	meter
Telemetry packet length (without header)	28	byte
Alarm packet length (without header)	14	byte
Telemetry transmission interval (periodic)	600	second (s)
Alarm transmission interval (random)	$\exp(\frac{1}{600})$	second
$T_{s}$ sub-frame duration	60	second
$T_{f}$ frame duration	600	second
Carrier frequency	EU 868	MHz
Number of uplink channels	3	−
Uplink channel frequencies	868.1, 868.3, 868.5 3	MHz
Bandwidth	125	kHz
Duty cycle	1%	−
Coding rate	4/5	−
Transmit power range	2–14	dBm
Beacon interval	60	second
Simulation time	86,400	second

**Table 5:** Telemetry packet delivery ratio and 95% confidence intervals for LoRaWAN under single-gateway deployment.

Distance (m)	100 Nodes		500 Nodes		1000 Nodes	
	Mean (%)	95% CI	Mean (%)	95% CI	Mean (%)	95% CI
100	100.00	— ^a^	99.72	[98.94, 100.00] ^b^	100.00	— ^a^
200	100.00	— ^a^	100.00	— ^a^	99.44	[97.89, 100.00] ^b^
300	100.00	— ^a^	99.16	[98.21, 100.00] ^b^	99.23	[97.90, 100.00] ^b^
400	100.00	— ^a^	98.42	[95.82, 100.00] ^b^	96.72	[93.41, 100.00] ^b^
500	100.00	— ^a^	97.85	[93.94, 100.00] ^b^	98.72	[97.77, 99.67]
600	99.58	[98.41, 100.00] ^b^	97.55	[94.91, 100.00] ^b^	96.52	[94.15, 98.89]
700	98.88	[96.98, 100.00] ^b^	98.21	[95.73, 100.00] ^b^	95.75	[90.93, 100.00] ^b^
800	99.16	[97.61, 100.00] ^b^	97.00	[93.92, 100.00] ^b^	95.05	[91.82, 98.28]
900	72.44	[67.11, 77.77]	63.54	[57.79, 69.29]	58.70	[51.45, 65.95]

Intervals are computed from five independent simulation runs using Student’s *t*-distribution with four degrees of freedom ($t_{0.025,4}=2.776$); half-width $=t\cdot s/\sqrt{5}$. ^a^ All five runs achieved 100% delivery; the sample standard deviation is zero, and the interval is undefined. ^b^ Upper bound truncated at 100% since PDR is bounded above by its physical maximum.

**Table 6:** Telemetry packet delivery ratio and 95% confidence intervals for RSrSF-LoRa under single-gateway deployment.

Distance (m)	100 Nodes		500 Nodes		1000 Nodes	
	Mean (%)	95% CI	Mean (%)	95% CI	Mean (%)	95% CI
100	100.00	— ^a^	99.72	[99.25, 100.00] ^b^	100.00	— ^a^
200	100.00	— ^a^	100.00	— ^a^	99.32	[99.26, 99.38]
300	100.00	— ^a^	99.24	[98.24, 100] ^b^	98.74	[98.35, 99.13]
400	99.72	[99.25, 100.00] ^b^	98.90	[97.15, 100] ^b^	97.90	[96.84, 98.96]
500	99.30	[98.07, 100.00] ^b^	97.92	[97.08, 98.76]	96.36	[94.11, 98.61]
600	99.44	[98.71, 100.00] ^b^	96.46	[94.21, 98.71]	94.72	[92.5, 96.94]
700	99.30	[98.43, 100.00] ^b^	96.04	[94.60, 97.48]	91.14	[89.39, 92.89]
800	99.10	[97.99, 100.00] ^b^	94.84	[93.15, 96.53]	90.77	[89.77, 91.77]
900	53.88	[50.11, 57.65]	55.28	[54.11, 56.45]	51.96	[47.24, 56.68]

Intervals are computed from five independent simulation runs using Student’s *t*-distribution with four degrees of freedom ($t_{0.025,4}=2.776$); half-width $=t\cdot s/\sqrt{5}$. ^a^ All five runs achieved 100% delivery; the sample standard deviation is zero, and the interval is undefined. ^b^ Upper bound truncated at 100% since PDR is bounded above by its physical maximum.

**Table 7:** Energy remaining in joules after 24 h for single-gateway scenario.

Nodes	LoRaWAN				RSrSF-LoRa			
	Alarms		Telemetry		Alarms		Telemetry	
	Min	Max	Min	Max	Min	Max	Min	Max
100	9999.34	9999.35	9999.00	9999.47	9998.24	9998.46	9999.08	9999.32
500	9999.14	9999.53	9998.67	9999.57	9997.76	9998.13	9999.04	9999.30
1000	9999.08	9999.55	9998.53	9999.64	9997.23	9998.07	9999.04	9999.32

**Table 8:** Telemetry packet delivery ratio and 95% confidence intervals for LoRaWAN under multi-gateway deployment.

Distance (m)	1000 Nodes		2100 Nodes	
	Mean (%)	95% CI	Mean (%)	95% CI
100	100.00	— ^a^	100.00	— ^a^
200	99.44	[98.68, 100] ^b^	99.44	[98.57, 100.00] ^b^
300	99.04	[97.40, 100.00] ^b^	99.16	[98.18, 100.00] ^b^
400	100.00	— ^a^	99.58	[98.32, 100.00] ^b^
500	99.76	[99.40, 100.12] ^b^	99.30	[98.43, 100.00] ^b^
600	99.88	[99.68, 100.00] ^b^	99.88	[99.68, 100.00] ^b^
700	99.66	[99.09, 100.00] ^b^	99.30	[97.80, 100.00] ^b^
800	99.02	[97.80, 100.00] ^b^	99.66	[99.19, 100.00] ^b^
900	99.96	[99.85, 100.00] ^b^	99.78	[99.17, 100.00] ^b^
1000	99.58	[98.40, 100.00] ^b^	99.68	[99.14, 100.00] ^b^
1100	100.00	— ^a^	98.88	[96.66, 100.00] ^b^
1200	100.00	— ^a^	98.08	[96.02, 100.00] ^b^
1300	99.66	[99.22, 100.00] ^b^	97.98	[95.60, 100.00] ^b^
1400	99.38	[98.44, 100.00] ^b^	98.06	[96.87, 99.25]

Intervals are computed from five independent simulation runs using Student’s *t*-distribution with four degrees of freedom ($t_{0.025,4}=2.776$); half-width $=t\cdot s/\sqrt{5}$. ^a^ All five runs achieved 100% delivery; the sample standard deviation is zero and the interval is undefined. ^b^ Upper bound truncated at 100% since PDR is bounded above by its physical maximum.

**Table 9:** Telemetry packet delivery ratio and 95% confidence intervals for RSrSF-LoRa under multi-gateway deployment.

Distance (m)	1000 Nodes		2100 Nodes	
	Mean (%)	95% CI	Mean (%)	95% CI
100	100.00	— ^a^	97.43	[94.18, 100.00] ^b^
200	99.16	[98.77, 99.55]	97.20	[97.09, 97.31]
300	99.48	[98.84, 100.00] ^b^	97.32	[96.71, 97.63]
400	99.38	[99.16, 99.60]	96.76	[94.43, 99.09]
500	99.24	[98.24, 100.00] ^b^	95.33	[90.91, 99.75]
600	99.44	[98.71, 100.00] ^b^	96.60	[92.40, 100.00] ^b^
700	98.10	[98.38, 99.82]	98.44	[98.12, 98.76]
800	98.96	[98.21, 99.71]	98.20	[97.37, 99.03]
900	99.32	[98.63, 100.00] ^b^	98.50	[98.13, 98.87]
1000	99.82	[99.44, 100.00] ^b^	98.35	[97.44, 99.26]
1100	98.46	[97.17, 99.75]	97.62	[96.76, 98.48]
1200	99.26	[99.15, 99.37]	97.12	[95.12, 99.12]
1300	98.28	[97.01, 99.55]	96.74	[94.64, 98.85]
1400	98.16	[96.74, 99.58]	94.96	[93.57, 96.35]

Intervals are computed from five independent simulation runs using Student’s *t*-distribution with four degrees of freedom ($t_{0.025,4}=2.776$); half-width $=t\cdot s/\sqrt{5}$. ^a^ All five runs achieved 100% delivery; the sample standard deviation is zero, and the interval is undefined. ^b^ Upper bound truncated at 100% since PDR is bounded above by its physical maximum.

**Table 10:** Energy remaining in joules after 24 h for seven-gateway scenario.

Nodes	LoRaWAN				RSrSF-LoRa			
	Alarms		Telemetry		Alarms		Telemetry	
	Min	Max	Min	Max	Min	Max	Min	Max
1000	9999.02	9999.43	9998.83	9999.39	9997.90	9998.25	9999.07	9999.84
2100	9999.20	9999.52	9998.81	9999.54	9997.66	9999.21	9999.02	9999.33

## Data Availability

All data associated with the research are available from the GitHub repository: https://github.com/SonileKM/Reliability-Evaluation-of-RSrSF-LoRa-and-LoRaWAN-for-Dense-Industrial-IoT-Networks-in-a-Smelter-Envi (accessed on 13 August 2026).
